# Triggering Receptor Expressed on Myeloid Cells-1 (TREM-1) in Inflammation and Disease: Mechanisms, Therapeutic Potential, and Future Directions

**DOI:** 10.3390/ijms262110386

**Published:** 2025-10-25

**Authors:** Neerja Trivedi, Jitendra D. Bhosale, Amit Pant, Sonali P. Suryawanshi, Prerna Tiwari, Peter W. Abel, Gopal P. Jadhav

**Affiliations:** 1 Department of Pharmacology and Neuroscience, School of Medicine, Creighton University, Omaha, NE 68178, USA; ntrivedi@nebraskamed.com (N.T.); jitendrabhosale@creighton.edu (J.D.B.); amitpant@creighton.edu (A.P.); prernatiwari@creighton.edu (P.T.); peterabel@creighton.edu (P.W.A.); 2 Department of Pharmacology, Bharati Vidyapeeth Deemed University Medical College, Pune 411043, Maharashtra, India; suryawanshi.sonali@bharatividyapeeth.edu

**Keywords:** TREM-1 modulators, soluble TREM1, biomarkers, inflammatory disorder, therapeutic interventions, PGLYRP-1, LPS, HMGB1, actin, eCIRP

## Abstract

Triggering receptor expressed on myeloid cells-1 (TREM-1), a member of the immunoglobulin superfamily, plays a crucial role in amplifying inflammatory responses, thereby contributing to the pathogenesis and progression of various inflammatory diseases. This review presents a comprehensive analysis of the current understanding of TREM-1 signaling and its dysregulation in disease pathology. Additionally, it explores the prognostic significance of TREM-1 across a spectrum of conditions. Targeting TREM-1 signaling represents a promising therapeutic approach for managing a wide range of diseases, including cancer, neurodegenerative disorders, cardiovascular diseases, and other inflammation-driven conditions. Previous reviews on TREM-1 have largely focused on its immunological role across diverse disease conditions and selective peptide-based inhibitors targeting its signaling pathway. However, recent discoveries have identified small-molecule modulators of TREM-1 that offer new opportunities for therapeutic intervention. Incorporating these findings would provide a more comprehensive and updated perspective on TREM-1 biology, particularly regarding its molecular regulation, drug-target potential, and translational relevance in inflammatory and immune-mediated disorders. Advances in this field are expected to be driven by structure-based drug design, particularly in the development of TREM-1 inhibitors. However, further research is needed to elucidate the predictive value of TREM-1 alterations and to evaluate them in prospective human studies prior to clinical decision-making.

## 1. Introduction

Inflammatory processes involve complex signaling mechanisms, with triggering receptor expressed on myeloid cells-1 (TREM-1) playing a pivotal role in amplifying immune responses. TREM-1, encoded by the *Trem-1* gene, belongs to the immunoglobulin superfamily and functions as a cell surface receptor that enhances inflammatory pathways through the release of cytokines and chemokines [1,2]. Activation of TREM-1 occurs in synergy with toll-like receptors (TLRs), G protein-coupled receptors, Fc receptors, and cytokine receptors, further intensifying proinflammatory signaling cascades [3,4]. Although the specific nature of putative ligands for TREM-1 remains elusive, various pathological conditions, such as bacterial and fungal infections, and ischemic stroke, have been shown to induce its expression [5,6,7]. Recent research indicates that dysregulated TREM-1 signaling contributes to the pathogenesis of several inflammatory diseases, such as sepsis, myocardial infarction, atherosclerosis, and ischemic stroke [8,9]. Additionally, TREM-1 upregulation has been implicated in non-alcoholic fatty liver disease [10], hepatocellular carcinoma, neurodegenerative disorders [11], and neonatal sepsis. A critical regulatory mechanism of TREM-1 involves its shedding via matrix metalloproteases (MMPs), particularly MMP-9, leading to the generation of its soluble form, *s*TREM-1 [12]. Alternatively, *s*TREM-1 can also arise from the translation of a splice variant of TREM-1 mRNA that lacks the transmembrane and cytoplasmic domains [13]. The presence of *s*TREM-1 in circulation is often proportional to disease severity, making it a promising biomarker for inflammatory conditions.

The TREM family comprises several receptors beyond TREM-1 and includes multiple receptors, such as TREM-2, TREM-3, and TREM-like transcripts TLT-1 and TLT-4, each with distinct immunological roles [13]. TREM-2 is the best characterized and exerts anti-inflammatory effects, particularly in microglia and macrophages. It enhances phagocytosis and suppresses pro-inflammatory signaling, contributing to neuroprotection in disorders like Alzheimer’s disease [14,15]. TLT-1 is mainly expressed in platelets and megakaryocytes, where it regulates hemostasis and modulates inflammation through interactions with the coagulation system [16]. TREM-3 has been described in mice, but its human homolog remains unclear [17]. TLT-4 is highly expressed in the spleen, especially in CD8α^+^ dendritic cells and specific macrophage subsets. It is associated with inflammatory pathologies such as sepsis, atherosclerosis, and lupus [13]. Collectively, TREM family receptors orchestrate diverse immune responses, reflecting their context-dependent roles in maintaining immune balance and contributing to disease pathogenesis.

Although existing reviews on TREM-1 have provided valuable insights into its pathophysiological role across a broad spectrum of inflammatory and infectious diseases, as well as its inhibition through peptide-based approaches, they remain largely limited in scope. Recent advances in small-molecule modulation of TREM-1 have received comparatively less attention. Given the expanding understanding of TREM-1–mediated immune amplification and its contribution to disease pathogenesis, integrating information on newly identified small-molecule TREM-1 modulators is necessary. Such compounds hold significant promise due to their favorable pharmacological properties, including greater stability, oral bioavailability, and ease of clinical translation compared with peptide inhibitors. A comprehensive information of these compounds would not only fill an important gap in the existing body of knowledge but also highlight their therapeutic potential as targeted anti-inflammatory agents in conditions characterized by pathological TREM-1 activation.

This review will provide a comprehensive analysis of TREM-1’s role in inflammatory signaling, its involvement in disease pathogenesis, and its potential as a diagnostic and prognostic biomarker. Furthermore, it will explore emerging therapeutic strategies targeting TREM-1, along with the challenges that must be addressed to translate these findings into clinical applications.

## 2. TREM-1 Signaling in Inflammation

**Figure 1:**
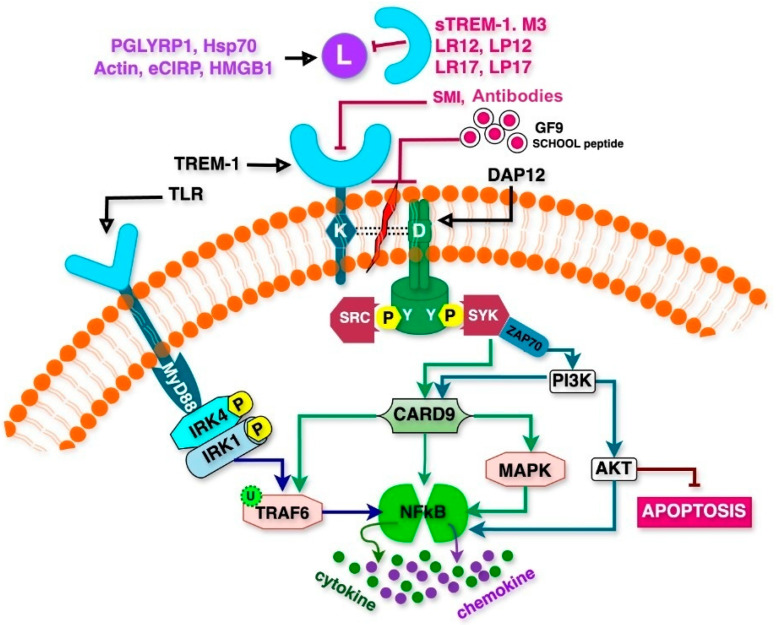
**TREM-1 signaling and inhibition approaches:** (A) TREM-1 signaling in inflammation (indicated as solid arrow lines in green, blue and black): Upon activation by ligands such as PGLYRP1, HSP70, actin, eCIRP, HMGB1, or LPS, TREM-1 associates with the adaptor protein DAP12, initiating an ITAM-mediated phosphorylation cascade through Src kinases, which activates SYK and ZAP70. This triggers downstream PI3K-AKT signaling that enhances inflammatory cell survival by inhibiting apoptosis, and the PI3K-ERK pathway to preserve mitochondrial integrity. Additionally, TREM-1 engages the CARD9–NFκB axis, contributing to neuroinflammation, and the CARD9–MAPK pathway, which is implicated in rheumatoid arthritis. Through synergistic interactions with Toll-like receptors (TLRs), TREM-1 amplifies inflammatory signaling via MyD88, PI3K, ERK1/2, and NFκB, ultimately promoting heightened production of pro-inflammatory cytokines and chemokines; (B) TREM-1 inhibition strategy (indicated as solid red lines): decoy receptors-*s*TREM-1, M3, LR12, LP12, LR17, LP17) act by competing with membrane-bound TREM-1 for ligand binding, thereby blocking its activation; SCHOOL peptide (GF9)-derived from the conserved transmembrane region of TREM-1, disrupts the interaction between TREM-1 and its adaptor protein DAP12; and small molecule inhibitors (SMIs)-bind with high affinity to the ligand-binding pocket of TREM-1, effectively blocking its activation.

Cell surface TREMs are found on immune cells including monocytes, neutrophils, macrophages, and dendritic cells. TREM-1, a 30 kDa glycoprotein receptor, comprises an immunoglobulin (Ig)-like V-type ectodomain, a transmembrane segment, and a short cytoplasmic domain lacking intrinsic signaling motifs. As shown in Figure 1 (see subheading A), to facilitate intracellular signaling, TREM-1 associates with DNAX activation protein 12 (DAP12) [8,11,18]. Upon activation, TREM-1 recruits DAP12 and triggers downstream signaling via the immunoreceptor tyrosine-based activation motif (ITAM). This interaction involves the electrostatic association of a positively charged lysine residue on TREM-1 with a negatively charged aspartic acid residue on DAP12. The phosphorylation of ITAMs permits the binding of Src homology 2 (SH2) domains, forming receptor complexes and leading to the activation of protein tyrosine kinases, such as Zeta-chain-associated protein kinase 70 (ZAP70) and Spleen Tyrosine Kinase (SYK). These kinases further activate the phosphoinositide 3-kinase (PI3K) and Akt pathways, promoting inflammatory cell survival by preventing apoptosis. Additionally, TREM-1-induced activation of PI3K and extracellular signal-regulated kinase (ERK) pathways sustains mitochondrial integrity and inhibits the pro-apoptotic factors BAD, BID, BAX, and cytochrome-C release, thereby enhancing cell survival [18].

TREM-1 functions synergistically with TLRs to mediate inflammatory responses. TLRs, which serve as key pathogen recognition receptors (PRRs), initiate inflammatory signaling upon detecting damage-associated molecular patterns (DAMPs) or pathogen-associated molecular patterns (PAMPs) [5,19,20]. TLR activation triggers the MyD88-dependent recruitment of IL-1 receptor-associated kinase-4 (IRAK-4), leading to MAP kinase activation and nuclear translocation of transcription factors such as AP-1, CREB, and NFκB, which subsequently regulate inflammatory gene expression [18,21,22]. Co-activation of TREM-1 and TLR4 results in the enhanced production of pro-inflammatory mediators via shared signaling pathways, including PI3K, IRAK1, ERK1/2, and NFκB. Notably, TREM-1 knockdown suppresses the expression of genes within the TLR4 pathway despite maintaining unaltered TLR4 levels, indicating that TREM-1 amplifies TLR-mediated inflammatory responses but is insufficient for prolonged inflammation independently [18,23,24].

The expression of TREM-1 is regulated by nuclear factor kappa-light-chain-enhancer of activated B cells (NFκB). In murine macrophage cell line RAW264.7, lipopolysaccharide (LPS) stimulation upregulates the p65 (RelA) subunit without affecting p50 expression. The p50/p50 homodimer negatively regulates TREM-1 transcription, whereas the p50/RelA heterodimer enhances its expression. Additionally, CCAAT/enhancer-binding protein alpha (C/EBPα) is crucial for basal TREM-1 transcription by activating the cAMP response element (CRE) site, independent of its phosphorylation status. CRE-binding protein (CREB) further promotes TREM-1 expression through phosphorylation by protein kinase A (PKA). Furthermore, activator protein-1 (AP-1), composed of ERK-activated c-Fos and JNK-activated c-Jun, positively regulates TREM-1 expression, whereas the transcription factor PU.1 acts as a negative regulator. RAW264.7 macrophages stimulated with LPS and *Pseudomonas aeruginosa* under PU.1 knockdown conditions exhibit increased TREM-1 expression. Additionally, nuclear factor erythroid 2-related factor 2 (Nrf2), upon activation by prostaglandins (PGs), downregulates TREM-1 expression. In experimental sepsis models, Nrf2-knockout mice exhibit heightened NFκB activation in response to LPS stimulation compared to control mice, further supporting the role of PG-induced Nrf2 activation in suppressing TREM-1 expression [18].

Soluble TREM-1 (*s*TREM-1) expression is regulated through the proteolytic shedding of its ectodomain by matrix metalloproteinases (MMPs), which are upregulated in response to bacterial products. MMP activation is driven by prostaglandin E2 (PGE2) as well as the PI3K, Akt, and NFκB signaling pathways. However, the precise mechanisms governing MMP-mediated TREM-1 shedding remain incompletely understood [18]. Nucleotide-binding oligomerization domain (NOD)-like receptors (NLRs) function alongside TREM-1 and TLRs in modulating inflammatory responses. NLRs (family) including NOD1/2 recognize microbial infections and sterile tissue damage, triggering inflammatory pathways [18]. NLRs activation in conjunction with TLR and TREM-1 signaling enhances NFκB-dependent cytokine production. Additionally, NOD1/2 detect cellular stress signals associated with hypoxia, endoplasmic reticulum (ER) stress, and nutrient deprivation, linking inflammatory diseases to ER stress [25,26]. Mechanistically, TREM-1 activation enhances NOD1/2 expression. It also increases NFκB signaling, thereby amplifying cytokine production and inflammation. Besides NOD1/2 priming, NLRs like NLRP3 form inflammasomes that activate caspase-1 to produce IL-1β and IL-18. In vitro studies show that TREM-1–DAP12 signaling can potentiate this pathway. The interactions between TREM-1, TLRs, and NLRs are complex and not yet fully understood. These mechanisms are discussed in Section 4.10 and need further investigation [18].

## 3. TREM-1 Activators and Their Role in Inflammatory Responses

As a key modulator of innate immunity and inflammatory responses, TREM-1 is a promising novel therapeutic target. Although the specific endogenous ligand/s of TREM-1 remain unclear, several pathogen-associated molecular patterns (PAMPs) and damage-associated molecular patterns (DAMPs) have been identified as potential activators. These ligands contribute to the amplification of inflammatory cascades, particularly in sepsis and other immune-related pathologies. Several endogenous and bacterial-derived molecules have been identified as putative ligands for TREM-1, each enhancing inflammation through direct or indirect interactions with TREM-1.

### 3.1. LPS Modulates TREM-1 Activation

LPS indirectly activates TREM-1 by binding to Toll-like receptor 4 (TLR4), which then interacts with TREM-1 to enhance immune responses [27,28]. TREM-1 is constitutively expressed in macrophages and is upregulated in response to LPS exposure. Studies on RAW264.7 macrophage-like cells have shown that *Trem-1* gene transcription is regulated by NFκB-1 and the cyclic AMP response element (CRE), both of which contribute to LPS-induced upregulation of TREM-1 promoter activity [27]. Research on human kidney-2 (HK-2) cells has demonstrated that TREM-1 activation promotes apoptosis while inhibiting autophagy following LPS stimulation [28]. Furthermore, in vivo studies using an LPS-induced endometritis mouse model revealed increased inflammatory cytokine secretion and an accumulation of polymorphonuclear neutrophils (PMNs) [29]. Additional findings by Zhu et al. confirmed that LPS-treated mice exhibited extensive PMN infiltration, leading to severe uterine tissue damage [30].

### 3.2. Peptidoglycan Recognition Protein 1 (PGLYRP1)

PGLYRP1 is an antimicrobial protein that forms complexes with bacterial membrane components such as peptidoglycan and LPS, leading to TREM-1 activation and subsequent oxidative stress and cell membrane depolarization. Additionally, PGLYRP1 has been reported to bind *s*TREM-1, suggesting a potential regulatory role [18,31,32].

### 3.3. High Mobility Group Box 1 (HMGB1)

HMGB1, a nuclear DNA-binding protein, functions as a DAMP in inflammatory conditions. It activates TLRs and the receptor for advanced glycation end products (RAGE), inducing inflammatory phenotypes both in vitro and in vivo. Studies on murine hepatocellular carcinoma models have demonstrated that HMGB1 binds to TREM-1 on necrotic hepatocytes, with an affinity (Kd) of =35.4 μM. This interaction triggers cytokine release in human THP-1 cells, highlighting its role in inflammation [33,34,35].

### 3.4. Actin

Actin, a fundamental cytoskeletal protein, is released extracellularly during necrosis and apoptosis, particularly in septic conditions. Elevated levels of extracellular actin can be cytotoxic to endothelial cells. Co-localization studies using confocal immunofluorescence microscopy have confirmed that actin binds to TREM-1 on platelets, further supporting its role as a TREM-1 ligand in sepsis models [35,36,37].

### 3.5. Heat Shock Protein 70 (Hsp70)

Hsp70 isoforms play a crucial role in protein homeostasis, including cellular protein folding, regeneration, and degradation. In necrotic cells, Hsp70 and HMGB1 collectively activate TREM-1, leading to an amplified pro-inflammatory response. Additionally, Hsp70 directly binds to TREM-1 on monocytes, resulting in upregulation of interferon-gamma (IFN-γ) and tumor necrosis factor-alpha (TNF-α) mRNA expression [35,38,39]

### 3.6. Cold-Inducible RNA-Binding Protein (eCIRP)

eCIRP, a DAMP, is widely elevated in the blood of patients suffering from hemorrhagic shock and sepsis. Under hypoxic conditions, macrophages stimulated with recombinant CIRP (rCIRP) release TNF-α and HMGB1, thereby enhancing inflammatory responses. Recent studies indicate that murine rCIRP exhibits high affinity (Kd = 120 nM) for recombinant murine TREM-1 (rmTREM-1), and inhibition of TREM-1 mitigates systemic and pulmonary inflammation in rCIRP-induced murine sepsis models [40,41].

### 3.7. CD177

CD177, also known as PRV-1 or NB1, is a glycosylphosphatidylinositol (GPI)-anchored protein expressed on a subset of human neutrophils [42]. CD177 directly interacts with TREM-1, amplifying immune responses through downstream signaling cascades. Additionally, an agonist antibody targeting TREM-1 (α-TREM-1) enhances wound healing and interleukin-22 (IL-22) production by promoting interactions between CD177^+^ neutrophils and macrophages [43]. In vitro studies have also demonstrated that anti-CD177 antibodies inhibit neutrophil transendothelial migration [44]. Understanding the CD177-TREM-1 axis is particularly relevant in inflammatory diseases such as inflammatory bowel disease (IBD), where dysregulated immune responses are common. Targeting this interaction may provide novel therapeutic avenues for disease management.

In summary, the identification of TREM-1 ligands is crucial for elucidating its role in inflammatory responses and developing novel therapeutic interventions. While LPS indirectly activates TREM-1 via TLR4, a range of endogenous and bacterial-derived molecules, including HMGB1, Hsp70, actin, eCIRP, PGLYRP1, and CD177, have been identified as putative ligands. Their interactions with TREM-1 contribute to immune activation, cytokine production, and disease progression, particularly in sepsis and other inflammatory disorders, as discussed in Table 1. Further research is needed to explore these ligand-receptor interactions, paving the way for targeted therapies in immune-mediated diseases.

## 4. TREM-1 Regulation in Inflammatory Disorders

As a major regulator of the inflammatory response, TREM-1 is frequently elevated in inflammatory conditions like sepsis, cancers, rheumatoid arthritis, CVDs, etc. In these conditions, TREM-1 increases cytokine synthesis and immune cell activation, which further intensifies inflammation. Control of inflammation is essential to avoid tissue damage and over activation of the immune system. TREM-1’s activity is modulated by mechanisms like the production of *s*TREM-1, which functions as a decoy receptor, and transcriptional regulation by NFκB and certain mRNAs. Herein, we discuss both acute and chronic inflammatory illnesses involving TREM-1 signaling and approaches to target it as a therapeutic to manage pathological inflammation.

### 4.1. TREM-1 Regulation in Sepsis and Neonatal Sepsis

**Figure 2:**
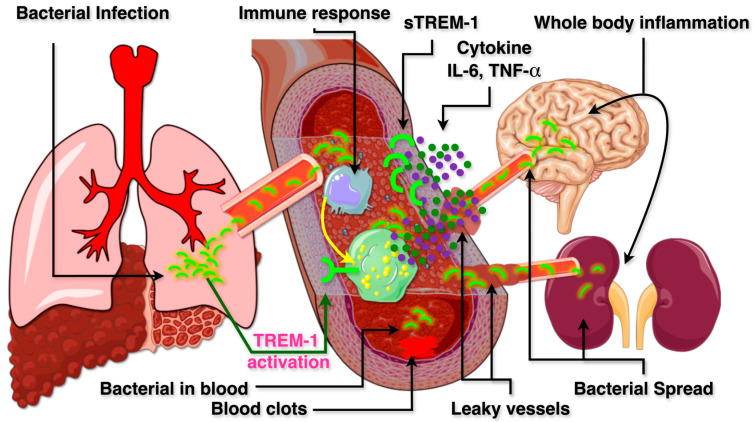
**TREM-1 signaling in sepsis**: Bacterial infection activates TREM-1 on neutrophils and monocytes, amplifying the production of pro-inflammatory cytokines including IL-1β, IL-6, TNF-α and *s*TREM-1. This cytokine surge exacerbates systemic inflammation, promoting bacterial dissemination and triggering a cascade that contributes to multiple organ failure characteristic of sepsis.

Detrimental TREM-1 activation in response to sepsis and neonatal sepsis is depicted in Figure 2. Sepsis is a life-threatening condition characterized by a dysregulated immune response to infection, leading to systemic inflammation, tissue damage, multiple organ failure, and high mortality rates. This pathological immune activation is driven by an excessive release of inflammatory cytokines such as tumor necrosis factor-alpha (TNF-α) and interleukin-1 beta (IL-1β), and IL-6, which contribute to vascular instability, hypotension, and multi-organ dysfunction.

TREM-1 has emerged as a critical modulator of the inflammatory response in sepsis. Studies have demonstrated that TREM-1 expression is significantly upregulated in neutrophils and monocytes from patients with bacterial infections, while its expression remains low in non-infectious inflammatory conditions. Furthermore, peritoneal neutrophils from patients with microbial sepsis exhibit increased TREM-1 expression, corroborating its role in sepsis-related immune dysregulation. Experimental models of LPS-induced septic shock in mice further support this finding, as TREM-1 upregulation was consistently observed. A key feature of TREM-1 activation is the release of *s*TREM-1, a circulating form of the receptor that serves as an indicator of immune activation. Elevated *s*TREM-1 levels have been detected in the serum and other biological fluids of both septic human patients and experimental animal models. Notably, in patients with ventilator-associated pneumonia, *s*TREM-1 levels in bronchoalveolar lavage fluid (BALF) accurately predicted the presence of bacterial or fungal infections. This suggests that *s*TREM-1 quantification above a critical threshold could serve as a reliable biomarker for disease severity in various inflammatory conditions. For instance, patients with septic shock exhibit markedly elevated *s*TREM-1 levels (814 pg/mL) compared to healthy individuals (1.77–135 pg/mL). Therapeutic inhibition of TREM-1 has shown promising results in experimental sepsis animal models. In LPS-induced septic mice, administration of a TREM-1 inhibitor (TREM-1/Fc fusion protein) resulted in a significant reduction in TNF-α and IL-1β production, ultimately decreasing mortality by 70%. These findings highlight the potential of TREM-1 as both a biomarker and a therapeutic target in sepsis [50,51,52].

Neonatal sepsis remains a significant global health challenge, contributing to high morbidity and mortality. The increased susceptibility of neonates to infections is largely attributed to their immature immune system, which is less effective in controlling microbial invasions. Neonatal sepsis often arises from bacterial infections originating in various organ systems, including the respiratory tract (pneumonia), urinary tract, gastrointestinal tract, and skin. When bacteria from these localized infections enter the bloodstream, the immune system initiates a strong inflammatory response. However, this uncontrolled immune activation leads to widespread inflammation, tissue injury, and vascular dysfunction. Increased vascular permeability results in leaky blood vessels and coagulation abnormalities, leading to hypotension and impaired oxygen delivery to vital organs. This ultimately contributes to neonatal morbidity and mortality. TREM-1 plays a crucial role in neonatal sepsis by amplifying the inflammatory response. Studies indicate that bacterial infections in neonates trigger TREM-1 activation in leukocytes, leading to an exaggerated secretion of inflammatory cytokines and *s*TREM-1. The release of *s*TREM-1 has significant correlation with the disease severity, suggesting that *s*TREM-1 could serve as a valuable diagnostic biomarker for neonatal sepsis as well. Importantly, experimental inhibition of TREM-1 in neonatal peripheral blood leukocytes has been shown to significantly reduce the secretion of pro-inflammatory cytokines. Thus, targeting TREM-1 therapeutically could provide a novel strategy for mitigating excessive inflammation and improve clinical outcomes in neonatal infections [41,51,53].

To summarize, in early sepsis, TREM-1 levels rise on monocytes and neutrophils. This amplifies TLR-driven cytokine release, such as TNF-α and IL-6, causing a cytokine storm. In neonates, the immature immune system makes this response more harmful. As sepsis worsens, surface TREM-1 declines while -*s*TREM-1 increases. *s*TREM-1 may act as a decoy receptor or signal immune imbalance. Late-stage sepsis often leads to immune paralysis. Blocking TREM-1 early improves survival in animal studies. In neonates, adjusting TREM-1 activity may help maintain defense without excessive inflammation.

### 4.2. TREM-1 Regulation in Cardiovascular Diseases (CVDs)

**Figure 3:**
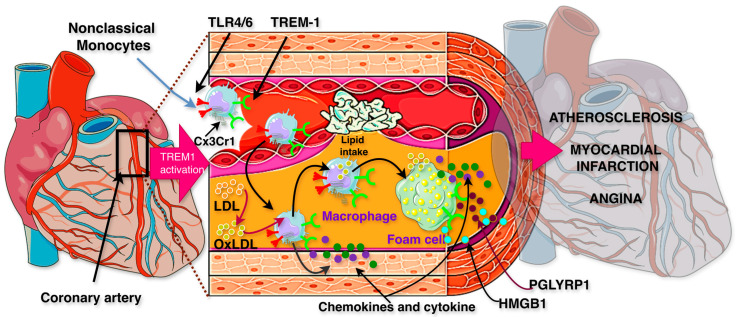
**TREM-1 signaling in CVDs:** Monocyte activation by TREM-1 binding to its ligand (HMGB1 or PGLYRP1) mediates SRC kinase-dependent DAP12 phosphorylation and SYK recruitment to activate downstream signaling molecules. Increased OxLDL activates monocytes, chronic inflammation induces recruitment and differentiation of monocytes to macrophages, which  enhances OxLDL uptake to form foam cells.

TREM-1 plays a critical role in CVDs by influencing myelopoiesis and inflammatory responses, as shown in Figure 3. The development of arterial atheroma and its instability are linked to TREM-1 overexpression. TREM-1 contributes to the expansion and instability of atherosclerotic plaques, which can be seen clinically as either coronary artery disease (CAD) or angina when the coronary is partially blocked or atherosclerotic myocardial infarction (AMI) when it is fully blocked. Additionally, TREM-1 is linked to an increased risk of endocarditis. Within the systemic circulation, TREM-1 skews hematopoietic differentiation towards monocyte production. In monocytes, transmembrane TREM-1 is activated by its ligands, including PGLYRP1, HMGB1, or LPS. This activation leads to the activation of NFκB, which in turn, promotes the transcription of genes encoding pro-inflammatory cytokines, chemokines, and various cell surface molecules. Additionally, TREM-1 synergizes with TLRs to amplify ILs and TNF-α release. Within atherosclerotic plaques, the upregulation of TREM-1 enhances monocyte transmigration into the intimal layer, where they encounter oxidized low-density lipoprotein (oxLDL). Upon ligand binding (e.g., PGLYRP1 from neutrophils or HMGB1 from macrophages), TREM-1 activation augments oxLDL uptake, promoting foam cell formation and contributing to atherogenesis. The activation of NFκB further drives the inflammatory response by translocating into the nucleus of macrophages, thereby perpetuating a pro-atherogenic environment. Pharmacological suppression of TREM-1 or epigenetic TREM-1 modification reduces atherosclerosis and the incidence of AMI in a mouse model with a notably low death rate, and it can also help individuals who are at risk of endocarditis inflammation [8,54,55,56,57].

Overall, in early atherosclerosis, TREM-1 is increased in vascular macrophages. It promotes foam cell formation, inflammation, and plaque instability. During acute MI, TREM-1 rises in infiltrating immune cells of the heart. This aggravates tissue injury and cardiac remodeling. *s*TREM-1 levels link with infarct size and predict poor outcomes. Blocking TREM-1 early after MI reduces infarct size and improves heart function. Its role in long-term heart failure is still uncertain. Thus, TREM-1 drives both chronic plaque progression and acute ischemic injury, offering stage-specific therapeutic targets.

### 4.3. TREM-1 Regulation in Neurological Disorders

Recent studies indicate that TREM-1 may also play a significant role in various neuroinflammation-related disorders, including subarachnoid hemorrhage, Parkinson’s disease, Alzheimer’s disease and ischemia. Neuroinflammation is a critical factor in the pathogenesis of subarachnoid hemorrhage (SAH) and is associated with worsened patient outcomes. Neuroinflammation worsens neuronal injury after a subarachnoid hemorrhage (SAH). It triggers harmful events such as the release of inflammatory cytokines and disruption of the blood–brain barrier. Microglia and other immune cells become activated, amplifying the inflammatory response. These processes cause neuronal death, brain swelling, delayed cerebral ischemia (DCI) and cerebral vasospasm (CVS). As a result, patients may develop long-term cognitive deficits and irreversible brain damage [58]. TREM-1 and *s*TREM-1 are key immune response factors in the brain after SAH. Studies have identified a strong correlation between plasma as well as cerebrospinal fluid (CSF) *s*TREM-1 levels and poor neurological scores, with elevated *s*TREM-1 levels observed in patients with initial severity of SAH. However, its role in specifically predicting CVS and DCI is not well-established. *s*TREM-1 may serve as a marker of inflammation and disease severity in SAH [59]. The pathophysiology of SAH involves the activation of TREM-1 pathways in both microglia and neutrophils. Microglial activation leads to a significant increase in the expression of neuroinflammatory genes, including IL-6, IL-1β, and mediator, TNF-α, as well as genes associated with the proinflammatory microglial phenotype (CD68, CD16, CD32, CD86).

**Figure 4:**
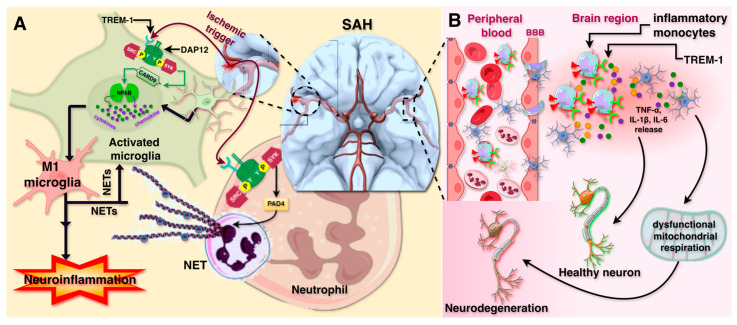
**Role of TREM-1 in Neuroinflammation and neurodegeneration in SAH:** (**A**) TREM-1 is activated through ischemic triggers, leading to the downstream activation of CARD9 and PAD4 pathways in neutrophils. Activated neutrophils create NETs, which further exacerbate neuroinflammation and cause secondary neuronal injury. (**B**) Ischemic triggers lead to activation of microglia, producing excessive proinflammatory cytokines, which subsequently impair BBB permeability, infiltration of peripheral immune cells and endothelial dysfunction, a major cause of neurodegeneration.

Concurrently, activated neutrophils form neutrophil extracellular traps (NETs), which exacerbate neuroinflammation, contribute to secondary neuronal injury, and compromise the integrity of the blood–spinal cord barrier (Figure 4B), a phenomenon also observed in spinal cord injury. TREM-1 activation further amplifies inflammation through its interaction with SYK, a key mediator of downstream proinflammatory signaling. Following SAH, both phosphorylated pSYK and non-phosphorylated SYK show increased expression (Figure 4A). Moreover, SAH activates the Card9-NFκβ pathway, leading to a substantial rise in the levels of Card9, pNFκβ, NFκβ, and PAD4-NETs, with significantly elevated PAD4 expression. These molecular changes collectively drive an aggressive inflammatory response that exacerbates neurological damage [59,60]. Importantly, therapeutic intervention targeting TREM-1 has shown promise in experimental in vitro and animal models. In vivo treatment with the TREM-1 inhibitory peptide (LP17) has been found to improve neurological function and mitigate neuronal injury in a mouse model of SAH. Inhibiting TREM-1 not only suppresses the transition of microglia to a proinflammatory state but also reduces NET formation and overall neuroinflammation. These findings suggest that TREM-1 could be a valuable therapeutic target for mitigating inflammation and improving outcomes in SAH patients.

In an MPTP-induced Parkinson’s disease (PD) mouse model, increased TREM-1 activity in peripheral monocytes accelerates the degeneration of dopaminergic neurons in the substantia nigra pars compacta (SNpc) due to sustained neuroinflammation. The involvement of TREM-1 in microglial activation suggests its potential to cross the blood–brain barrier (BBB). Suppressing TREM-1 through genetic deletion or pharmacological inhibition (via LP17) protects neurons by reducing innate immune responses and monocyte infiltration. Interestingly, in the absence of TREM-1, a subset of monocytes (Ly6C^+^/CX3CR1^+^) with possible neuroprotective functions emerges, indicating a shift toward reparative macrophages [11,61].

Alzheimer’s Disease (AD) is characterized by amyloid-beta (Aβ) plaque deposition and tau pathology, contributing to neuroinflammation and synaptic impairment. TREM-1 has emerged as a key regulator of microglial activity in AD. Elevated TREM-1 expression in AD-associated microglia enhances phagocytosis while simultaneously amplifying inflammation. It facilitates microglial recruitment and activation by recognizing Aβ aggregates. Wilson et al. highlight the critical role of TREM-1 in microglial immunometabolism and its impact on neuronal function during aging and AD. TREM-1 activation in microglia amplifies neuroinflammation, disrupts metabolism, and impairs mitochondrial function, contributing to cognitive decline. TREM-1 signaling through DAP12 sustains NFκB activation, leading to increased secretion of proinflammatory cytokines such as TNF-α and IL-1β, thereby intensifying neurotoxicity. Genetic variations in TREM-1 (e.g., rs6910730G) are linked to reduced Aβ clearance and accelerated disease progression. In postmortem AD mice brains, TREM-1 colocalizes with microglia around amyloid plaques and correlates with disease severity. Notably, *Trem1*-deficiency in mice prevents age-related metabolic decline, inflammation, and memory loss, rescuing ribose 5-phosphate levels. *Trem1*-deficient microglia resist amyloid-β42-induced bioenergetic disruption. In 5XFAD mice, *Trem1* haploinsufficiency preserves memory, microglial homeostasis, and reduces neuritic dystrophy. In APPSwe mice, *Trem1* deficiency restores synaptic mitochondrial function, glucose uptake, and prevents memory decline. Thus, TREM-1 promotes cognitive decline in aging and AD, highlighting its role in disease pathogenesis and its therapeutic potential [62,63,64,65,66].

Ischemic stroke or cerebral ischemia: TREM-1 also plays a significant role in cerebral ischemia [67]. TREM-1 expression is elevated in microglia, as well as infiltrating monocytes. This upregulation exacerbates neuroinflammation by promoting the release of proinflammatory cytokines such as TNF-α and IL-1β, which worsen neuronal damage. Additionally, TREM-1 increases BBB permeability, facilitating the infiltration of peripheral immune cells and enhancing the expression of matrix MMP-9, which contributes to endothelial dysfunction and hemorrhagic transformation in stroke [68]. Elevated serum levels of *s*TREM-1 have been observed in patients with acute ischemic stroke and correlate significantly with stroke severity. This relationship suggests that *s*TREM-1 may serve as a useful biomarker for evaluating the extent of neuroinflammation and neuronal damage following ischemic events [69]. Inhibiting TREM-1 with synthetic peptides like LP17 has been shown to reduce neuronal damage, enhance hippocampal synaptic plasticity, decrease infarct size, reduce BBB leakage, and improve functional recovery in ischemic stroke models. These findings support TREM-1 inhibition as a promising therapeutic strategy for alleviating ischemic brain injury [70].

To assess TREM-1’s role in overall neurological disorders, it should be noted that after ischemic stroke or CNS injury, TREM-1 levels rise in microglia and infiltrating macrophages. It boosts cytokine release and disrupts the blood–brain barrier (BBB), intensifying neuroinflammation. During the acute phase, this increases neuronal damage. In chronic disorders like Alzheimer’s disease, ongoing TREM-1 activation keeps microglia active and drives neurodegeneration. Blocking TREM-1 in stroke models lowers infarct size, BBB leakage, and neuron loss. Thus, TREM-1 worsens acute brain injury and may fuel chronic neurodegeneration if left unchecked.

### 4.4. TREM-1 Regulation in Rheumatoid Arthritis

Rheumatoid arthritis (RA) is a chronic autoimmune inflammatory disease affecting approximately 1–2% of the global population. Although the exact etiology remains unclear, RA is thought to arise from a complex interaction between genetic susceptibility and environmental factors. The disease is characterized by an aberrant immune response, in which the immune system mistakenly targets synovial joints, resulting in persistent inflammation, synovial hyperplasia, and progressive destruction of cartilage and bone. TREM-1 has emerged as a crucial regulator of inflammation in RA. It influences adaptive immunity, where in synergy with PAMP ligands, TREM-1 enhances the expression of CD40, CD86, and MHC Class II on monocytes, facilitating their differentiation into immature dendritic cells and promoting T-cell activation. TREM-1 activation also has an effect on innate immunity. Increased infiltration and activation of macrophages play a central role in RA pathogenesis. Notably, macrophage colony-stimulating factor (M-CSF or CSF-1) dependent cells are widely used in the collagen-induced arthritis (CIA) model of RA. Recent studies have demonstrated a significant upregulation of *Trem-1 mRNA*, with a 6.5-fold increase in human RA synovial samples and a remarkable 165-fold elevation in CIA-affected paws compared to control tissues. Furthermore, increased expression of functional TREM-1 at the protein level has been confirmed in both human RA synovial samples and the CIA model. To assess the functional relevance of TREM-1 in RA, cells isolated from RA patient knee joints via radiofrequency ablation were cultured, revealing an immediate secretion of TNF-α, IL-8, IL-1, and granulocyte-macrophage CSF (GM-CSF). Notably, this cytokine release was significantly amplified upon TREM-1 activation, underscoring its role in driving inflammatory responses.

**Figure 5:**
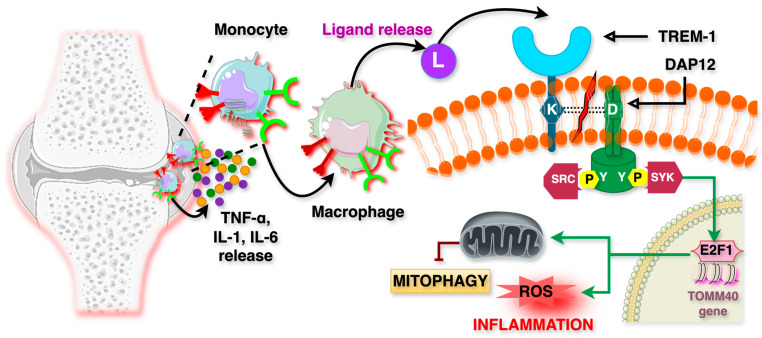
**TREM-1 signaling in rheumatoid arthritis:** In inflamed synovium, TREM-1 activation promotes IL-1, IL-6, and TNF-α release, driving macrophage polarization. Epigenetically, TREM-1 enhances TOMM40 expression via pSYK-dependent activation of E2F1, which suppresses PINK1-PARKIN-mediated mitophagy.

Mechanistically, Activation of the E2F1/mitophagy axis contributes to mitochondrial dysfunction and enhances inflammatory signaling in synoviocytes and infiltrating immune cells. Modulating this pathway can attenuate disease severity in the CIA model, suggesting a mechanistic link between cell cycle regulation, mitochondrial quality control, and autoimmune pathology. In RA synovial fluid, macrophage polarization triggers the activation of TREM-1, which subsequently leads to the release and accumulation of proinflammatory cytokines and reactive oxygen species (ROS) within the inflamed synovium. Upon activation, TREM-1 significantly enhances the expression of the transcription factor translocase of the outer mitochondrial membrane 40 (TOMM40) in macrophages. This occurs through a pathway involving p-SYK-mediated activation of the E2F1 gene, followed by its nuclear translocation, as illustrated in Figure 5. The increased expression of TOMM40 and E2F1 contributes to the suppression of PINK1-PARKIN-mediated mitophagy. Additionally, elevated E2F1 levels play a further role in promoting inflammatory responses within macrophages. Impaired mitochondria also produce excess intracellular ROS through the oxidative phosphorylation cascade involving ADP and ATP. Notably, inhibiting TREM-1 in the CIA animal model significantly reduced synovial inflammation, cartilage damage, and diminished joint swelling. This inhibition also limits the activation of the NFκB pathway, which is central to the inflammatory response. These findings suggest that TREM-1 inhibition could provide a novel therapeutic approach to manage RA, particularly in patients who do not respond to traditional therapies targeting TNF-α or other cytokines. However, these findings require validation in human rheumatoid arthritis to confirm clinical relevance and therapeutic potential [71,72,73,74].

### 4.5. TREM-1 Regulation in Periodontitis

**Figure 6:**
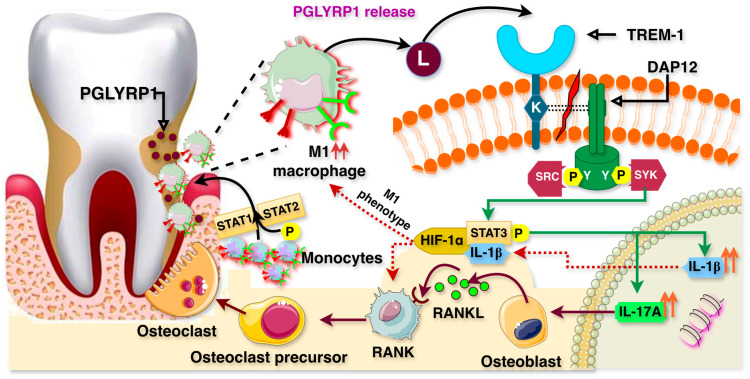
**Mechanistic pathway depicting TREM-1–mediated inflammatory signaling in ligature-induced periodontitis:** PGLYRP1 from plaque/bacterial infection serves as ligand to activate the TREM-1 signaling in neutrophils and macrophages. This initiates epigenetic activation of TREM-1-PGLYRP1-IL1β signaling pathway with downstream phosphorylation cascades, involving SYK, SRC, activating STAT3 and promoting nucleated IL1β and IL17A expression. Concurrently, IL-17A induces increase in RANKL/OPG ratio, leading to dysregulated bone homeostasis. STATs activation and PGLYRP1-induced inflammation contribute to dysregulated bone resorption and alveolar bone loss.

Periodontitis primarily arises from the accumulation and progression of dental plaque, a dense microbial biofilm composed predominantly of bacteria that adhere to the tooth surface. If left untreated, plaque accumulation triggers an inflammatory response that ultimately leads to the degradation of periodontal tissues. Studies have demonstrated that TREM-1 is expressed in various biological fluids and tissues of individuals with periodontitis, including saliva, serum, gingival tissues, and gingival crevicular fluid. Upon microbial stimulation, TREM-1 expression is significantly upregulated, contributing to periodontal disease progression. In vivo studies using a murine model of periodontitis have highlighted the critical role of TREM-1 in alveolar bone resorption. The receptor activator of NFκB (RANK)-receptor activator of NFκB ligand (RANKL)-osteoprotegerin (OPG) signaling axis is well established in osteoclast maturation, bone modeling, and remodeling. Under physiological conditions, RANKL and OPG function in a complementary manner to regulate bone homeostasis, with the RANKL/OPG ratio favoring osteoclastogenesis. However, in a ligature-induced periodontitis model (Figure 6), TREM-1 activation increased the RANKL/OPG ratio, potentially through the upregulation of IL-17A [71,75,76,77,78,79]. Epigenetically, the long non-coding RNA (LncR) ANRIL, found on human chromosome 9p21, is a genetic risk factor for several diseases, including atherosclerosis, periodontitis, diabetes, and cancer (APDC). Its ortholog in mice, LncR-APDC, located on chromosome 4, plays a regulatory role in periodontitis progression. The TREM-1-PGLYRP1-IL1β signaling pathway becomes activated in response to bacterial biofilm formation and its removal in periodontitis. In APDC-deficient macrophages and neutrophils, IL1β release increases, suggesting enhanced communication among these immune cells, as well as with T cells, epithelial cells, and fibroblasts. Additionally, the interaction between PGLYRP1 and TREM-1 is highly active in the gingival tissue of APDC-KO models, particularly between epithelial cells, macrophages, and neutrophils. Consequently, the extreme activity of the TREM-1-PGLYRP1-IL1β pathway in APDC deficiency leads to a more rapid and excessive immune response to pathogen stimulation, resulting in severe periodontal inflammation and increased bone loss [80].

Moreover, work by Wu et al. supports the role of TREM-1 in promoting periodontal inflammation by driving M1 macrophage polarization. The newly identified STAT3-HIF1α-IL1β signaling pathway, acting downstream of TREM-1, plays a key role in regulating M1 polarization in periodontitis. Activation of TREM-1 triggers STAT3 phosphorylation via the JAK2 signaling pathway, and the activated STAT3 subsequently enhances HIF-1α expression. In periodontitis, the macrophage M1/M2 ratio significantly increases, while TREM-1 inhibition primarily suppresses M1 polarization [76,81,82]. Notably, pharmacological inhibition of TREM-1 using the LP17 peptide resulted in a reduced RANKL/OPG ratio, downregulation of IL-17a, and a significant decrease in bone loss. These findings highlight TREM-1 inhibition as a promising therapeutic approach for periodontitis.

### 4.6. TREM-1 in Hepatic and Renal Ischemic Injury

TREM-1 also plays a significant role in other ischemic conditions including renal ischemia and hepatic ischemia–reperfusion (I/R) injury. In hepatic I/R injury, TREM-1 plays a crucial role in driving inflammatory responses. It has been reported that targeting TREM-1 through genetic knockout or pharmacological inhibition reduces liver damage by mitigating inflammation and leukocyte recruitment. For example, in mouse models of hepatic ischemia, TREM-1 deficiency or blockade led to a reduction in proinflammatory cytokine expression and enhanced liver function following reperfusion [49].

In a similar vein, renal ischemic injury upregulates TREM-1 expression, particularly in the S3 segment of proximal tubules in the cortico-medullary region. This increase in TREM-1 expression contributes to inflammation and tissue damage, consistent with its role in immune responses across multiple organ systems during ischemic conditions. Although therapeutic approaches aimed at modulating TREM-1 in murine models reduced inflammatory responses, these treatments did not prevent renal injury, leukocyte infiltration, or organ dysfunction [24].

### 4.7. TREM-1 Regulation in Hepatic Fibrosis

**Figure 7:**
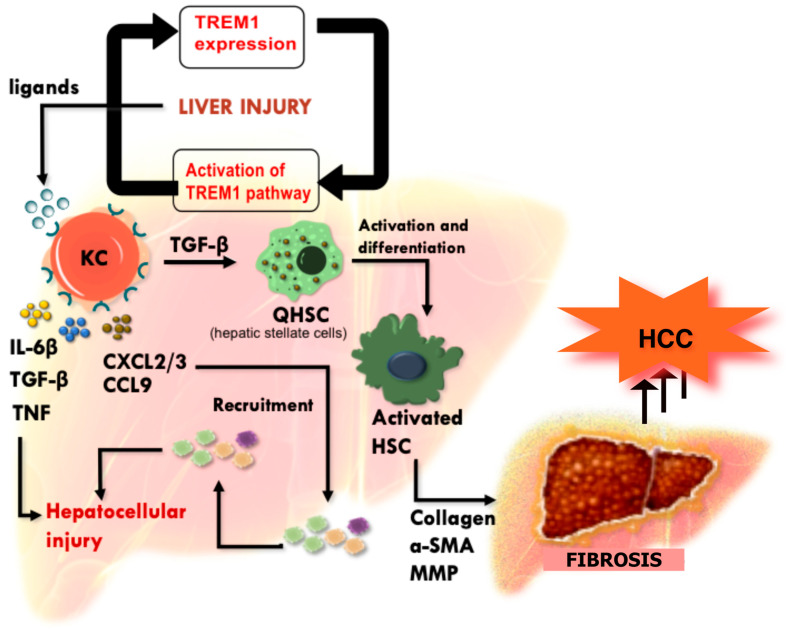
**TREM-1 signaling in Liver Fibrosis:** Chronic liver injuries release HMGB1, HSP70, and PGLYRP (endogenous ligands), which activate TREM-1 on the surface of Kupffer cells to secrete pro-inflammatory cytokines TGF-β1, ILs, and chemokines (CCL9). TGF-β1 activates and differentiates quiescent HSCs into activated HSCs, leading to liver fibrosis.

Hepatic fibrosis arises from chronic liver injury, during which persistent inflammation activates hepatic stellate cells (HSCs) to produce excess extracellular matrix. Hepatocellular carcinoma (HCC) is a malignancy strongly linked to chronic inflammation, where persistent infiltration of inflammatory cells contributes to hepatocellular damage, leading to liver fibrosis and cirrhosis. Emerging evidence suggests that TREM-1 plays a crucial role in amplification of inflammation and fibrogenesis, as shown in Figure 7. Elevated TREM-1 expression in human tumor-activated hepatic stellate cells (HSCs) has been correlated with poor survival outcomes in HCC patients, highlighting its potential as a key driver of disease progression. In carbon tetrachloride (CCl_4_)-induced mouse models, TREM-1 is implicated at multiple stages of HCC development, from the activation of Kupffer cells to the modulation of pro-fibrogenic pathways and the progression of liver injury and fibrogenesis. Herein, TREM-1 is highly expressed on the surface of Kupffer cells and monocyte-derived macrophages, where it is activated by its endogenous ligands (for example, HMGB1, HSP70, and PGLYRP). Ligands are released in response to chronic liver injury, triggering Kupffer cells to secrete pro-inflammatory cytokines such as transforming growth factor-beta 1 (TGF-β1) and interleukins, alongside murine chemokines such as CCL9. The latter plays a vital role in recruiting bone marrow-derived monocytes/ macrophages to sites of liver damage, whereas TGF-β1 primarily mediates the activation and differentiation of quiescent HSCs into activated HSCs. This transition is a critical step in liver fibrosis, as activated HSCs contribute to extracellular matrix remodeling through excessive collagen deposition, α-smooth muscle actin (α-SMA) release, and matrix metalloproteinase (MMP) secretion [83,84,85].

Given its fundamental role in fibrosis in complicating HCC pathophysiology, targeting TREM-1 represents a promising therapeutic strategy for managing HCC and liver fibrosis. Studies have demonstrated that silencing the *Trem1* gene (*Trem1^−/−^*) significantly attenuates liver fibrosis progression. Additionally, blocking TREM-1 activity suppresses the migration of HCC cells during the early stages of carcinogenesis, particularly in response to conditioned media (CM) from HSC-derived cancer-associated myofibroblasts (CAMFs). Furthermore, pharmacological inhibition of TREM-1 has been shown to reduce inflammation and lipid accumulation in diet-induced nonalcoholic fatty liver disease (NAFLD) models. These findings collectively underscore the potential of TREM-1 targeted therapies in mitigating liver fibrosis and HCC progression, paving the way for novel therapeutic interventions [83,84,85].

### 4.8. TREM-1 Regulation in Chronic Obstructive Pulmonary Disease (COPD)

COPD is a progressive respiratory disorder characterized by obstructive airflow resulting from chronic airway inflammation and alveolar destruction. Clinically, it manifests as dyspnea, persistent cough with sputum production, and wheezing. The main etiological factor is long-term exposure to cigarette smoke, although environmental pollutants and occupational particles also contribute to disease onset and progression. In COPD, alveolar macrophages (AMs) exhibit aberrant activation, producing elevated levels of pro-inflammatory cytokines and proteolytic enzymes that exacerbate tissue injury. Concurrently, peripheral blood monocytes are excessively recruited to the pulmonary, where they differentiate into inflammatory macrophages that further amplify cytokine release, including IL-6. Despite the availability of current therapeutic options such as bronchodilators, corticosteroids, and smoking cessation, these interventions only alleviate symptoms and slow disease progression, emphasizing the need for novel disease-modifying targets.

Emerging evidence implicates TREM-1 as a pivotal mediator of chronic inflammation and immune dysregulation in COPD. Studies have demonstrated upregulated TREM-1 expression in both AMs and circulating monocytes of COPD patients. Cigarette smoke exposure directly induces TREM-1 expression and indirectly via smoke-derived exosomes. Elevated levels of *s*TREM-1 correlate with impaired lung activity, specifically reduced forced expiratory volume in one second (FEV_1_), indicating a relationship between TREM-1 activation and disease severity. Mechanistically, cigarette smoke promotes macrophage polarization toward a pro-inflammatory M1 phenotype, wherein TREM-1 synergizes with NLRP3 activation and pyroptosis, driving alveolar injury and extracellular matrix remodeling [86,87]. Experimental studies have shown that TREM-1 inhibition attenuates pro-inflammatory cytokine production, suppresses macrophage activation, and mitigates lung tissue destruction, underscoring its pathogenic role. Although clinical data remain limited, accumulating evidence positions TREM-1 as both a biomarker of disease progression and a potential therapeutic target for modulating macrophage-driven inflammation during stable and exacerbation phases of COPD.

### 4.9. TREM-1 Regulation in Inflammatory Bowel Disease (IBD)

IBD is a chronic autoimmune disorder that causes inflammation in the gastrointestinal tract. The two main forms are Crohn’s disease and ulcerative colitis. Current treatments can reduce symptoms and induce remission, but IBD remains a lifelong condition with no cure. This highlights the need for new therapeutic targets. Recent research identifies TREM-1 as a key driver of intestinal inflammation in IBD. TREM-1 is highly expressed on macrophages, neutrophils, and dendritic cells in inflamed intestinal tissue. It amplifies immune responses in synergy with TLRs. This interaction increases the release of pro-inflammatory cytokines such as TNF-α, IL-6, and IL-1β, which disrupt the epithelial barrier and promote chronic mucosal injury. Levels of *s*TREM-1 are often elevated in the blood of IBD patients. However, these levels do not clearly distinguish between active and remission stages of disease. Importantly, high TREM-1 expression in circulating monocytes has been linked to poor response to anti-TNF therapy, suggesting its role as a biomarker for treatment prediction. In animal models of colitis, blocking or knocking out TREM-1 reduces inflammation and protects the intestinal barrier [88,89,90,91].

### 4.10. TREM-1-TLR-NLR Network Synergy Regulation

The TREM-1-TLR-NLR forms a tightly coordinated regulatory network that governs innate immune responses during infection, tissue injury, and chronic inflammation. TLRs, such as TLR4 via LPS, trigger intracellular signaling cascades leading to NF-κB activation and cytokine synthesis. Concurrently, TLR4 stimulation upregulates TREM-1 expression on myeloid cells, including neutrophils and macrophages, where TREM-1 functions as a critical amplifier that potentiates cytokine release, particularly TNF-α, IL-6, and IL-1β via the DAP12–Syk signaling [3,92,93]. This synergy between TLR4 and TREM-1 initiates the priming (Signal 1) and activation (Signal 2) required for NLRP3 inflammasome activation. Signal 1 induces transcription of NLRP3 and pro-IL-1β, while Signal 2, mediated by cellular stress signals such as ROS, ATP, and mitochondrial DNA, promotes caspase-1 activation and the maturation of IL-1β and IL-18. TREM-1 further facilitates this process by enhancing ROS production and inflammasome activation [94]. Collectively, this synergistic TREM-1-TLR-NLR network forms a feed-forward inflammatory loop, wherein each component amplifies the activity of the others, leading to robust inflammatory responses [95]. Although crucial for host defense, dysregulation of this network contributes to excessive inflammation observed in sepsis, COPD, IBD, and ischemia–reperfusion injury. Therefore, therapeutically, targeting one node, particularly TREM-1, can dampen the entire inflammatory circuit without completely disabling host immunity, making it an attractive intervention point.

REM-1, TLR4, and NLRs (especially NLRP3) often cooperate in inflammatory signaling, but their interaction hierarchy and priority vary depending on: Disease type and stage (acute vs. chronic), Cell type involved (monocyte, neutrophil, epithelial, etc.), Triggering ligand (e.g., LPS, DAMPs, viral RNA, crystals) and Tissue context (e.g., gut, lung, brain, liver). Table 2 explains a summary of their functional interactions and hierarchy in major diseases.

## 5. TREM-1 Modulation: A Potential Avenue for Inflammatory Disease Therapy

TREM-1 modulation is implicated in various pathological conditions, where the downregulation of TREM-1 signaling at the genetic and protein expression levels of ligands or receptors could lead to significant improvements in TREM-1-related inflammatory disorders. TREM-1 has been identified as a key regulator of pro-inflammatory innate immune responses in both infectious and non-infectious diseases, including CVD, atherosclerosis [57], multiple cancers, and sepsis. Therefore, targeted intervention in TREM-1 signaling at physiological and genetic levels may provide a promising therapeutic approach to mitigate these inflammatory conditions.

### 5.1. Genetic Ablation of TREM-1

Extensive studies using single knockout (KO) mouse models such as *Trem-1^−/−^*, and *Treml-1*; double KO model such as *Trem-1/3^−/−^*, and combined KO model such as *Trem-1^−/−^ Apoe^−/−^* have highlighted the central role of TREM-1 in modulating inflammation, immune cell trafficking, and disease progression in a range of pathological conditions.

*Trem-1^−/−^* mice consistently exhibit attenuated inflammatory responses across multiple models, including bacterial (*Leishmania*, *Legionella*, *Klebsiella*), viral (*LCMV*, *influenza*), parasitic, and autoimmune diseases, without compromising microbial clearance in most cases. These mice show decreased neutrophil infiltration, lower cytokine production, and reduced tissue damage, confirming the pro-inflammatory and immunopathogenic role of TREM-1.

In *Treml1^−/−^* mice, impaired platelet aggregation and enhanced susceptibility to polymicrobial infections underscore the importance of triggering receptor expressed on myeloid cells-like transcript 1 (TLT-1) in maintaining hemostatic balance and leukocyte function.

Combinatorial models further emphasize pathological relevance of TREM-1. In the *Trem-1^−/−^*-*Apoe^−/−^* model, TREM-1 deficiency significantly mitigates atherosclerosis by reducing monocytosis, inflammatory plaque formation, and expression of chemokine receptors like CX3CR1 on nonclassical monocytes. Similarly, in the context of *Trem-1^−/−^* with angiotensin-II induced abdominal aortic aneurysm, inflammation and aortic wall degradation were markedly reduced, which was associated with decreased expression of Il1b, TNF-α, MMP2, and MMP9. In liver pathology, *Trem-1* deletion dampens Kupffer cell activation and fibrosis, while restoration of TREM-1 in these cells reverses protection, confirming its cell-specific role.

The double KO *Trem-1/3^−/−^* model has elucidated the essential function of TREM-1 in transepithelial neutrophil migration, with significant implications in pneumonia and reproductive tract infections. In cancer models, *Trem-1* deficiency led to reduced tumor growth (melanoma, fibrosarcoma), less immunosuppressive activity of MDSCs and higher expression of PD-1 on CD8^+^T cells, pointing to a role in shaping the tumor immune microenvironment.

Collectively, findings across these genetic models reveal that TREM-1 is a potent amplifier of innate immune responses and a driver of immunopathology, particularly in chronic inflammation, cardiovascular disease, infectious models, and cancer. These insights support the therapeutic targeting of TREM-1, not only in acute infections and sepsis but also in inflammation-driven conditions such as atherosclerosis, fibrosis, and tumor progression. Knockout mouse models (*Trem-1^−/−^* and/or *Trem-3^−/−^*) have been utilized to investigate TREM-1 functions across various disease contexts, as summarized in Table 3.

TLT-1 serves as a natural inhibitor of TREM-1, conferring anti-inflammatory effects. Notably, LR17 (or LP17), a 17-amino-acid peptide derived from the extracellular region of TLT-1, acts as a potent TREM-1 inhibitor [96]. TREM-1 activation enhances pro-inflammatory responses, promoting pathogen clearance during microbial infections.

**Table 3:** Insights from TREM-1 targeted genetic mouse models.

Gene	Key Findings	Ref.
*Trem-1^−/−^*	TREM-1 plays a crucial role in bacterial translocation and systemic inflammation. *Trem-1^−/−^* mice infected with *Leishmania major* or *influenza* virus exhibited reduced lesion sizes, lower neutrophilic infiltration, and decreased morbidity, while maintaining effective pathogen control comparable to wild-type (WT)controls.	[97,98]
	*Trem1*^−/−^ mice reduced hepatocellular tumorigenesis induced by diethylnitrosamine. It attenuated Kupffer cell activation by downregulating IL-6, IL-1β, TNF, CCL2, and CXCL10, and inhibited liver injury by modulating key inflammatory pathways (p38, ERK1/2, JNK, MAPK, and NF-κB).	[83]
	TREM-1 has been shown to play a role in viral hepatitis immunopathology, mostly through neutrophil activity. *Trem1^−/−^* mice cleared viral hepatitis faster than wild-type mice and had less liver inflammation and damage. *Trem1^−/−^* mice infected with *Lymphocytic Choriomeningitis Virus* (LCMV) produced less CCL2 and TNF-α in their livers compared to wild-type mice.TREM-1 has been implicated in the immunopathology of viral hepatitis, primarily through the activity of neutrophils. *Trem1^−/−^* mice cleared viral hepatitis more quickly than wild-type mice and exhibited reduced liver inflammation and damage. Furthermore, *Trem1^−/−^* mice infected with LCMV showed lower hepatic levels of CCL2 and TNF-α compared to wild-type mice.	[99]
	In *Leishmania major*, influenza virus, and *Legionella pneumophila* infection models of colitis, *Trem1^−/−^* animals displayed reduced morbidity and immune-mediated pathology without impairing microbial clearance.	[98]
	Trem-1 KO mice were found to be more vulnerable to oral infection with *Klebsiella pneumoniae* due to translocation in the small intestine. Mice infected with *Klebsiella pneumoniae* liver abscess (KPLA) exhibited higher mortality rates. This highlights the critical role of TREM-1 signaling in defending the host against bacterial infections and supporting effective mucosal immunity in the small intestine. Overall, Trem-1-KO mice displayed increased bacterial dissemination, liver and systemic inflammation, and higher mortality.	[97]
	TREM-1 plays a pivotal role in inflammation-driven intestinal carcinogenesis. In the wild-type *Trem1^+/+^* mouse model treated with azoxymethane (AOM) and dextran-sodium sulfate (DSS), TREM-1 was present in tumors but absent from the surrounding tumor-free mucosa. This suggests that TREM-1 mediates the recruitment of neutrophils into colon cancers and colitis-associated malignancies in the AOM/DSS model. The lack of TREM-1 signaling in *Trem1^−/−^* mice significantly reduces the development of intestinal tumors.	[100]
	TREM-1, an important regulator of Kupffer cell activation, triggers persistent liver inflammatory responses, activates hepatic stellate cells, and promotes liver fibrosis. *Trem1^−/−^* mice mitigated hepatic inflammation, fibrosis, and Kupffer cell activation. Restoring *Trem1*-sufficient Kupffer cells reversed these effects.	[101]
	TREM-1 regulates neutrophil migration and reactive oxygen species (ROS) generation via NOX2. TREM-1 phosphorylates protein kinase B (AKT), a signaling enzyme that regulates cell migration and survival. *Trem-1^−/−^* mice neutrophils displayed defective chemotaxis and reduced AKT phosphorylation.	[102]
	In the angiotensin II-induced abdominal aortic aneurysm (AAA) mouse model, AngII enhances TREM-1 production via AngII receptors, which affects monocyte mobilization from the spleen and infiltration into the aortic wall. Consequently, TREM-1 activation promotes monocyte penetration into the aorta and triggers a proinflammatory response, exacerbating AAA severity. *Trem1^−/−^* (*Apoe^−/−^Trem1^−/−^*) mice exhibited reduced AAA progression and severity by decreasing aortic inflammation and lowering the expression of Il1b, Tnfa, Mmp2, and Mmp9 mRNA, along with reducing macrophage content.	[103]
	Activation of the TREM-1 receptor intensifies the inflammatory response to hepatic ischemia/reperfusion (I/R), as indicated by increased blood levels of organ damage and inflammatory markers. In a hepatic I/R model, *Trem-1^−/−^* mice exhibited lower levels of tissue injury markers, inflammatory mediators, and neutrophil infiltration compared to wild-type mice. Additionally, *Trem-1^−/−^* mice demonstrated improved survival, suggesting that TREM-1 suppression could have significant therapeutic potential for hepatic I/R. The protective effects in *Trem-1^−/−^* mice were evident through reductions in injury markers, cytokines/chemokines, neutrophil infiltration, apoptosis, and overall tissue damage.	[49]
	*Trem-1^−/−^* mice tumor models exhibited TREM-1’s therapeutic potential in cancer. *Trem-1^−/−^* mice showed slower growth of melanoma (B16F10) and fibrosarcoma (MCA205) tumors. More single-cell RNA-Seq and functional experiments of *Trem-1^−/−^* tumor infiltrates indicated that myeloid-derived suppressor cells (MDSCs) have a lower immunosuppressive potential and higher PD-1 expression on CD8+ T cells.	[104]
*Endo Trem-1* ^−/−^	TREM-1 modulates vascular inflammation and endothelial cell (EC) activation, influencing inflammatory cell mobilization. *Trem1^−/−^* mice exhibited improved vasoreactivity, protection from septic shock, and increased survival.	[105]
*Trem-1 KO CRISPR*	TREM-1 plays a crucial role in intracerebral hemorrhage (ICH)-induced brain damage, leading to significant behavioral impairment and mortality. In mice, *Trem-1* knockdown using CRISPR improved neurobehavioral outcomes compared to ICH+ control CRISPR mice. *Trem-1* knockdown mice exhibited reduced TREM-1 expression in microglia after ICH, along with decreased IL-1β levels and an increase in anti-inflammatory factors such as M2 (Arg-I).	[106]
*Trem-1* ^−/−^ *.lpr*	*Trem1^−/−^* mice with lupus-prone backgrounds exhibited elevated serum BAFF, anti-dsDNA antibody levels, expanded lymphocyte populations, and increased renal immune complex deposition, leading to severe lupus symptoms and higher mortality.	[107]
*Tremligand1 (Treml1*^−/−^)	*Treml1^−/−^* mice exhibited increased plasma cytokine levels and mortality compared to WT mice. Platelet aggregation was impaired, leading to excessive bleeding and localized inflammatory lesions. These mice were more susceptible to polymicrobial infections, highlighting the role of TLT-1 in leukocyte activation.	[16,96,108]
*Trem1/3* ^−/−^	In a *Trem-1/3^−/−^* mouse model of *Pseudomonal pneumonia*, TREM-1 facilitates neutrophil migration across airway epithelial cells in the lungs, thereby intensifying inflammation. Exposure to *Pseudomonas aeruginosa* led to increased mortality, accompanied by higher local and systemic cytokine production. Despite exhibiting normal bacterial killing, phagocytosis, and chemotaxis, these neutrophils demonstrated reduced infiltration into the airways. While they successfully migrated through endothelial monolayers, they were unable to traverse airway epithelial barriers formed at the air-liquid interface.In a *Trem-1/3^−/−^* mouse model of *Pneumococcal pneumonia*, exposure to *S. pneumoniae* resulted in increased mortality and enhanced bacterial growth at the infection site. The infection triggered a rapid infiltration of TREM-1-positive neutrophils into the bronchoalveolar region, while macrophages, which inherently express high levels of TREM-1, remained unaffected. *Trem-1/3^−/−^* mice exhibited a significantly reduced innate immune response in the airways upon *S. pneumoniae* challenge, characterized by delayed neutrophil recruitment and reduced phagocytic activity of alveolar macrophages.In a mouse model of *Chlamydia trachomatis* genital infection, neutrophils contribute to tissue damage in the female reproductive tract, specifically the uterus and oviduct, as part of the adaptive immune response. Consistent with previous findings, TREM-1/3 facilitates transepithelial neutrophil migration in the uterus and uterine glands. However, *Trem-1/3^−/−^* mice did not show a significant reduction in chlamydial burden or genital tract infection compared to control mice. Notably, *Trem-1/3^−/−^* animals exhibited significantly less hydrometra in the uterine horns, fewer uterine glands infiltrated by polymorphonuclear cells, and elevated levels of granulocyte colony-stimulating factor (G-CSF). Additionally, these mice displayed reduced degradation of the luminal epithelium.	[109,110,111]
*Trem-1^−/−^ Apoe^−/−^*	TREM-1 aggravates atherosclerosis. In the *Apoe^−/−^* animal model under dyslipidemic conditions, TREM-1 expression is markedly elevated in circulating and lesion-infiltrating myeloid cells. *Trem1^−/−^ Apoe^−/−^* animals subjected to dyslipidemia exhibited a 40% reduction in aortic atherosclerosis, along with decreased skewed monocytes and mitigated monocytosis. Nonclassical monocytes in these mice showed significantly lower Cx3cr1 expression compared to their *Trem1^+/+^ Apoe^−/−^* counterparts. Additionally, the thoracoabdominal aorta displayed a less inflammatory plaque profile, characterized by a substantial reduction in macrophage density and necrotic core size.	[55,57]

### 5.2. Peptide/Protein Molecules as TREM-1 Modulators

TREM-1 inhibitory peptides/proteins, discussed in detail below, are molecules designed to suppress TREM-1 signaling and mitigate excessive inflammatory responses associated with various diseases, including sepsis, ischemia–reperfusion injury, rheumatoid arthritis, and cancer. These inhibitors function by blocking TREM-1 activation, disrupting its interaction with its signaling adaptor DAP-12, or acting as decoy receptors. Table 4 depicts the sequences and physiological functions of TREM-1 inhibitory proteins/peptides. Their mechanisms of TREM-1 inhibition are illustrated in Figure 1 (see inhibitory approach B).

***s*****TREM-1**: *s*TREM-1 is thought to function as a decoy receptor for TREM-1 binding to TREM-1 ligands and inhibiting TREM-1 activation and the associated release of pro-inflammatory cytokines [18].

**Dodecapeptide LR12**: The most well-known TREM-1 inhibitory peptide, LR12, also known as Nangibotide, is a TREM-1 antagonist derived from amino acids 94 to 105 in TLT-1. LR12 is a natural TREM-1 inhibitor that behaves similarly to *s*TREM-1. It competes with TREM-1 for ligand binding, reducing aortic inflammation during abdominal aortic aneurysm [103]. It limits cardiac inflammation by decreasing the recruitment of neutrophils and movement of classical monocytes to the heart [54]. In adult mini-pigs, LR12 injection decreases sepsis-induced cardiovascular dysfunction and organ failure, thus displaying positive benefits during septic shock. LR12 therapy significantly minimizes pathogenic damage, as well as neutrophil infiltration and activity. It inhibits pulmonary edema and LPS-induced production of pro-inflammatory cytokines and chemokines. In mice with acute lung injury, LR12 promotes the release of anti-inflammatory cytokines by decreasing TREM-1 expression, boosting *s*TREM-1 release, and blocking the NFκB signaling pathway. LR12 may also be effective for regulating the excessive inflammatory response that occurs with periodontal inflammation. As previously stated, TREM-1 synergizes with TLRs to activate monocytes for inflammatory responses. Human primary monocytes are activated with *Porphyromonas ginigivalis* LPS by increasing TREM-1 and *s*TREM-1 mRNA levels, as well as producing IL-1β, TNF-α cytokines and IL-8 chemokines. LR12 suppresses the activation of these macrophages to some extent [112].

**Safety Profile of LR12 (Nangibotide)**: Preclinical studies conducted in mice, pigs, and non-human primates demonstrated that LR12 exhibits a favorable safety profile, with no evidence of toxicity or immunosuppression. Importantly, pathogen clearance remained unaffected, indicating preserved innate immune competence. LR12 conferred protection against hyperinflammatory responses in various disease models, including sepsis, myocardial infarction, colitis, and ischemia–reperfusion injury. Moreover, there was no indication of delayed wound healing or increased susceptibility to infection.

In clinical evaluations, the Phase I trial in healthy volunteers confirmed excellent tolerability, with no serious adverse events, dose-limiting toxicities, or signs of cytokine dysregulation or immune suppression. In the Phase IIa ASTONISH trial involving patients with septic shock, nangibotide was well tolerated across a dose range of 0.3–1.5 mg/kg/h. The incidence of adverse events was comparable between treatment and placebo groups, and there was no increase in secondary infections or sepsis-related complications. Notably, treatment was associated with improvements in organ function parameters, suggesting potential therapeutic benefit.

In summary, LR12 appears safe and well-tolerated across both preclinical and clinical studies, effectively modulating inflammatory responses without compromising host defense mechanisms. Ongoing Phase III trials aim to further confirm its long-term safety and efficacy in critically ill patient populations.

**17 aa peptides LR17 and LP17**: LR17s are formed from amino acids 94 to 108 in hTLT-1 or mTLT-1, whereas LP17s are derived from a highly conserved region in the hTREM-1 or mTREM-1 extracellular domains. They both act as decoy peptides for TREM-1 activation. LR17 was demonstrated to be helpful in a polymicrobial sepsis mouse model. LR12-treated mice recovered from polymicrobial sepsis with considerably enhanced bacterial clearance, entirely preventing septicemia and increasing survival rates. Administration of LR17 during experimental sepsis provides partial protection against organ failure of the liver, lung, and kidney [108]. Feng and colleagues [11] found that TREM-1 induced learning and memory deficits in an LPS-challenged rat model. Treatment with the TREM-1 inhibitor LP17 dramatically suppresses the LPS-induced proinflammatory cascade, increases autophagy-related proteins, and improves cognitive function [11].

**SCHOOL peptide GF9**: Since the exact structure of the endogenous TREM-1 ligand(s) remains unidentified, novel ligand-independent TREM-1 inhibitors have been developed using an immune signaling approach known as the Signaling Chain HOmoOLigomerization (SCHOOL) method. This method describes the signal transduction mechanism in multichain immune recognition receptors (MIRRs), which occurs through the homooligomerization of receptor and adaptor subunits. As a MIRR family member, TREM-1 is noncovalently associated with the signaling adaptor DAP-12 in the membrane. According to the SCHOOL model, ligand binding to TREM-1 induces dimerization or multimerization of the TREM-1/DAP-12 complex, leading to DAP-12 homooligomerization and subsequent receptor activation. Short synthetic peptides with receptor-specific sequences can target the membrane interactions of MIRR subunits involved in ligand recognition and signal transduction. One such inhibitory peptide, GF9, is designed to disrupt TREM-1/DAP-12 interactions, thereby preventing DAP-12 homooligomer formation and alleviating inflammation-related disorders in animal models. GF9, composed of nine amino acids derived from the highly conserved transmembrane core region of both human and murine TREM-1, has demonstrated significant therapeutic potential. In xenograft models of human non-small cell lung cancer, GF9 effectively inhibits tumor growth. Furthermore, in vivo administration of GF9 one hour before LPS exposure significantly improves survival in murine models of LPS-induced septic shock. In vitro treatment with GF9 in J774 murine macrophages markedly reduces LPS-induced cytokine production, including TNF-α, IL-1β, and IL-6 [73,113]. GF9 has also shown efficacy in treating collagen-induced arthritis by preserving cartilage and bone integrity [73]. Additionally, GF9 significantly decreases serum levels of macrophage colony-stimulating factor (M-CSF or CSF-1), a key factor promoting tumor growth and metastasis, indicating its potential anticancer properties via M-CSF suppression.

To overcome GF9’s limitations, such as its short half-life and lack of cell selectivity in vivo, a synthetic high-density lipoprotein (HDL)-based delivery system has been proposed to target macrophages and extend peptide stability. While free GF9 at a dose of 2.5 mg/kg is ineffective, HDL-bound GF9 exhibits therapeutic efficacy at the same dose in treating collagen-induced arthritis in mice [73]. These findings highlight GF9 as a promising TREM-1 inhibitor with potential applications in inflammatory diseases and cancer therapy.

**Safety Profile of GF9**: Preclinical investigations in murine models of sepsis, colitis, and liver injury demonstrated that GF9 effectively inhibits TREM-1/DAP12 interactions, thereby attenuating excessive cytokine release while maintaining phagocytic capacity and bacterial clearance. Notably, GF9 administration did not increase mortality in infected animals, indicating preserved immune competence. There were no signs of systemic toxicity, organ dysfunction, or histopathological abnormalities across treatment groups.

Although GF9 has not yet progressed to human clinical trials, available preclinical evidence indicates a strong safety profile with no toxic or immunosuppressive effects. Nonetheless, comprehensive toxicological, pharmacokinetic, and first-in-human studies are required to fully establish its clinical safety and translational potential.

**TREM-1-Fc-protein**: The TREM-1-Fc fusion protein functions as a TREM-1 neutralizing agent, and it has been used to detect the presence of TREM-1 ligands in sepsis patient sera. TREM-1 expression is significantly upregulated on neutrophils and macrophages during *Pseudomonas aeruginosa*-induced peritonitis, which may be associated with increased mortality. Inhibition of TREM-1 signaling using the TREM-1-Fc fusion protein has been shown to prolong survival in animal models of *P. aeruginosa*-induced peritonitis by blocking TREM-1-mediated immune activation. However, rather than affecting macrophage phagocytic function in vitro, TREM-1 suppression primarily attenuates production of key pro-inflammatory cytokines, including IL-1β, TNF-α, MCP-1, and IFN-γ [114].

Additionally, the presence of circulating TREM-1 ligands in sepsis patient sera has been demonstrated using the TREM-1-Fc fusion protein. In a subset of sepsis samples, patient sera activate monocytes ex vivo, and this response is specifically blocked by TREM-1-Fc, indicating that soluble or cell-associated TREM-1 ligands contribute to monocyte activation in these cases. However, the precise identity and phenotype of the circulating TREM-1 ligand remain undefined [115]. Collectively, these findings highlight the utility of the TREM-1-Fc fusion protein as both a functional probe for ligand detection and a potential diagnostic tool in inflammatory diseases.

**Table 4:** Peptide modulators of TREM-1 and their physiologic effects.

Inhibitor	Origin	Amino Acid Sequence	Physiological Effects	Ref.
hLR12	hTLT-1	LQEEDAGEYGCM	Attenuates myocardial inflammation, mitigates atherosclerosis progression, prevents vascular dysfunction and inflammation, modulates excessive inflammatory response in periodontal disease, and decreases TREM-1 dimerization on monocytes and neutrophils. It also promotes hepatocyte regeneration in TAA-induced ALF and reduces aortic inflammation in AAA.	[28,54,55,103,105,112,116]
mLR12	mTLT-1	LQEEDTGEYGCV		
hLR17	hTLT-1	LQEEDAGEYGCMVDGAR	Prevents septicemia and improves survival rates. Functions as a TREM-1 inhibitory peptide derived from TLT-1.	[108,117]
mLR17	mTLT-1	LQEEDTGEYGCVVEGAA		
hLP17	hTREM-1	LQVEDSGLYQCVIYQPP	Inhibits LPS-induced proinflammatory cascades and provides protective effects in sepsis models.	[11,118]
mLP17	mTREM-1	LQVTDSGLYRCVIYHPP		
hGF9	hTREM-1	GFLSKSLVF	Reduces LPS-induced TNF-α, IL-1β, and IL-6 in J774 murine macrophages in vitro and inhibits TREM-1/DAP12 interaction at the transmembrane level.	[73,113]
mGF9	mTREM-1	GLLSKSLVF		
TREM-1-Ig-Fc fusion protein		Lowers TNF-α and IL-1β serum levels, serves as a diagnostic and prognostic tool, and neutralizes TREM-1 while activating monocytes in sepsis patients.		[114,115]
CD177		Anti-CD177 antibody inhibits trans-endothelial migration of neutrophils. CD177 modulates neutrophil migration via integrin and chemoreceptor regulation, downregulates membrane-bound proteinase-3 expression, and interacts with endothelial cells to regulate inflammation.		[42,68,119]

### 5.3. Small Molecule TREM-1 Modulators

**Figure 8:**
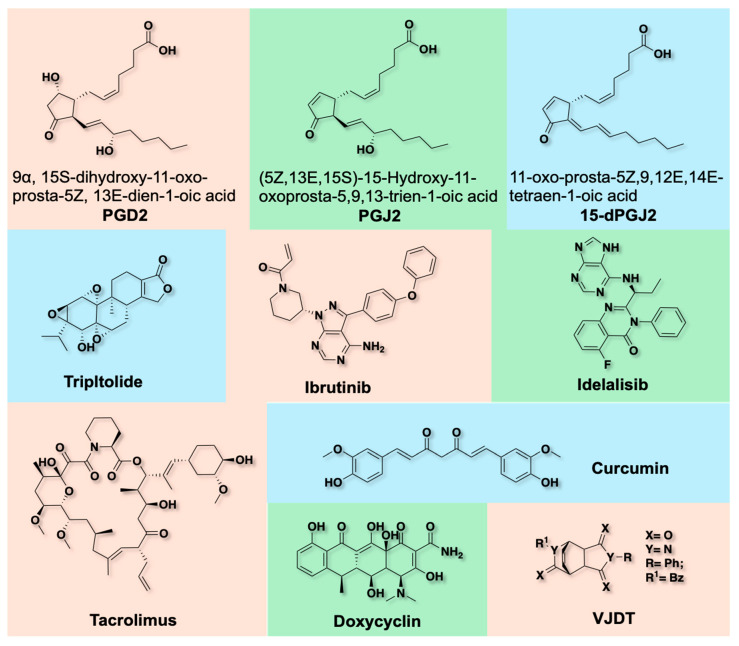
List of small molecules that interfere with the expression of TREM-1, including PGD2, PGJ2, 15-*d*PGJ2, Triptolide, Ibrutinib, Idelalisib, Tacrolimus, Curcumin, Doxycycline and VJDT.

TREM-1 has emerged as a crucial modulator of innate immunity and inflammatory responses. While therapeutic strategies targeting TREM-1 have primarily been restricted to preclinical animal models, recent research has explored the development of peptides, mimetics, and small-molecule inhibitors to regulate TREM-1 activity. Inhibiting TREM-1 signaling has protective effects in a variety of inflammatory conditions, including inflammatory arthritis [72,120,121], inflammatory bowel disease [90], pancreatitis [122], sepsis [5,19,108], myocardial inflammation [54], hemorrhagic shock, and acute ischemia–reperfusion [123]. Among the most promising TREM-1 inhibitors, LR12 peptide has shown significant survival benefits in animal models of sepsis and is currently undergoing Phase 2 clinical trials. However, due to its short half-life (~2–3 min) and due to proteolytic hydrolysis, its clinical application remains limited. This underscores the need to develop small-molecule inhibitors of TREM-1 with improved pharmacokinetic and pharmacodynamic properties. Given the absence of FDA-approved TREM-1 inhibitors, recent research has focused on identifying small molecules capable of modulating TREM-1 expression and activity, offering potential breakthroughs in the treatment of sepsis, inflammatory diseases, and immune disorders. Figure 8 depicts chemical structures of small molecules that have been shown to inhibit TREM-1 and Figure 1 (see inhibitory approach B) provides mechanism of TREM-1 inhibition by small molecules.

**PGD2, PGJ2, and 15-*d*PGJ2**: PGD2, PGJ2, and 15-*d*PGJ2 are naturally occurring prostaglandin derivatives characterized by cyclopentanone ring structures. Extensive in vitro and in vivo studies have demonstrated that their physiological functions and anti-inflammatory effects are primarily mediated through PGD2 receptors (DP1 and DP2 receptor types). However, the precise mechanisms underlying their immunomodulatory effects remain incompletely understood, suggesting that these molecules have additional effects on immune signaling pathways. A study by Syed et al. provided novel insights into the immunoregulatory properties of PGD2, PGJ2, and 15-*d*PGJ2 in the innate immune response. The findings indicate that at high concentrations (10 µM), these lipid mediators downregulate TREM-1 expression in macrophages. Furthermore, the study revealed that PGJ2 exerts its inhibitory effects on TREM-1 by activating NF-E2-related factor-2 (Nrf2) while partially suppressing NFκB signaling and contributing to its immunosuppressive properties [124].

**Triptolide**: Triptolide, an active compound from *Tripterygium wilfordii* Hook F, is known for its immunosuppressive and anti-inflammatory properties and is traditionally used to treat rheumatoid arthritis (RA). RA patients exhibit elevated TREM-1 levels in synovial fluid and *s*TREM-1 in plasma, which correlate with disease severity, indicating the pivotal role of TREM-1 in RA inflammation. Fan et al. [81] investigated whether triptolide’s anti-RA effects are mediated through TREM-1 inhibition. Bioinformatics analysis identified TREM-1 signaling as a key pathway influenced by triptolide.

In a collagen-induced arthritis (CIA) rat model, triptolide suppressed TREM-1 signaling, reducing TREM-1 expression in ankle joint tissues and *s*TREM-1 in synovial fluid. Further in vitro experiments showed that triptolide decreased TREM-1 and DAP12 levels, inhibited JAK2/STAT3 activation, and reduced pro-inflammatory cytokines (TNF-α, IL-1β, IL-6) in LPS-stimulated U937 cells. These findings suggest that triptolide may exert its anti-inflammatory effects in RA by inhibiting the TREM-1 signaling pathway [81,125].

**Ibrutinib**: Ibrutinib is an irreversible Bruton’s tyrosine kinase (BTK) inhibitor that impedes B-cell proliferation and survival, and is commonly used in the treatment of various malignancies, including mantle cell lymphoma, chronic lymphocytic leukemia, and Waldenstrom’s macroglobulinemia. BTK plays a crucial role in innate immunity, as it is expressed not only in B cells but also in polymorphonuclear neutrophils (PMNs) and macrophages, where it is particularly involved in TREM-1 signaling in monocytic cells. Recent work by Stadler et al. [126,127] demonstrated that ibrutinib inhibits TREM-1-mediated neutrophil activation. In an in vitro study, PMNs isolated from healthy donors exhibited an oxidative burst when stimulated with TREM-1-specific or control monoclonal antibodies. At high concentrations (100 nM), ibrutinib inhibited the phorbol ester (PMA)-induced oxidative burst, while at lower doses (3 nM), it completely blocked the TREM-1 ligation-induced oxidative burst. Additionally, ibrutinib was shown to nearly fully suppress the phosphorylation of ERK1/2 downstream of TREM-1 activation, indicating a higher selectivity for TREM-1 over TLR4. These findings were further validated in ex vivo and in vivo models, where PMN stimulation and animals pretreated with ibrutinib supported the results. Overall, these data suggest that BTK inhibition via ibrutinib diminishes TREM-1-mediated PMN function, providing a potential clinical strategy for modulating immune responses driven by TREM-1, such as those involved in sepsis, inflammatory bowel disease, peripheral artery disease, and psoriasis [126,127].

**Idelalisib**: Idelalisib is a phosphatidylinositol 3-kinase (PI3K) inhibitor used in the treatment of non-Hodgkin lymphoma. Similarly to Bruton’s tyrosine kinase (BTK), PI3K is crucial for TREM-1-mediated immune activation. Alflen et al. investigated the role of PI3K in TREM-1 and TLR-mediated neutrophil activation. The study assessed the effects of idelalisib on TLR or TREM-1-mediated activation of polymorphonuclear neutrophils (PMNs), both with and without idelalisib treatment, as well as ex vivo stimulation of PMNs from blood samples of patients undergoing idelalisib therapy. The results demonstrated that idelalisib selectively inhibits TREM-1-mediated neutrophil functions, including oxidative burst, degranulation, L-selectin shedding, and IL-8 release, by suppressing TREM-1-associated signaling downstream of PI3K. Importantly, the impaired neutrophil activation observed in treated patients highlights the clinical relevance of these findings [128,129].

**Tacrolimus (FK506)**: Tacrolimus, a macrolide compound, is used as an immunosuppressive adjunct, often in conjunction with other medications, to prevent organ rejection in kidney transplant recipients, wherein the recipient’s immune system attacks the transplanted organ. Originally, tacrolimus was identified as an antifungal agent, displaying weak activity against *Aspergillus fumigatus*. As an anti-inflammatory agent, tacrolimus inhibits the expression of pro-inflammatory cytokines, reduces enzyme activity, and lowers levels of inflammatory mediators.

Recent studies on fungal infections have highlighted the critical role of TREM-1, which is highly expressed in infected tissues. Additionally, TREM-1 activation, induced by *Pseudomonas aeruginosa*, exacerbates inflammation in corneal keratitis. Consequently, Huang et al. [130] investigated whether the antifungal and immunosuppressive effects of tacrolimus are mediated through the inhibition of TREM-1. They first confirmed that TREM-1 expression was elevated in the corneas of patients with fungal keratitis compared to normal corneas, and similarly, in a murine model of *Aspergillus fumigatus* keratitis. Furthermore, they examined TREM-1 suppression by tacrolimus in both in vitro *RAW264.7* macrophage cells and an in vivo murine model of *Aspergillus fumigatus* keratitis. In vitro experiments involved the activation of *RAW264.7* cells with zymosan and subsequent treatment with tacrolimus. Results from PCR and ELISA analyses demonstrated a significant reduction in TREM-1 expression in tacrolimus-treated *RAW264.7* cells compared to controls following zymosan stimulation. The in vivo study aimed to determine if tacrolimus could mitigate ocular damage following *Aspergillus fumigatus* infection. Animals were administered subconjunctival injections of tacrolimus or a vehicle control. The findings revealed that tacrolimus treatment reduced corneal damage in the early stages of infection, decreased TREM-1 expression in infected corneas, and attenuated the expression of inflammatory factors. Tacrolimus did not alter fungal growth in the context of fungal keratitis. This suggests that its protective effect on the cornea occurs through modulation of host responses rather than antifungal activity. Tacrolimus may reduce corneal damage in the early stages of infection by downregulating TREM-1 expression. Additional anti-inflammatory mechanisms are also likely involved in this protective effect [130,131].

**Curcumin**: Curcumin (diferuloylmethane) is a naturally occurring phytophenolic compound derived from the rhizome of *Curcuma longa* Linn., responsible for the characteristic yellow pigmentation of turmeric, a widely used culinary spice. In traditional Indian Ayurvedic medicine, curcumin has been employed for its therapeutic properties in managing inflammatory and infectious conditions. Notably, curcumin crosses the blood–brain barrier, where it exerts anti-inflammatory effects by suppressing the activation of astrocytes and microglia, as well as promoting the polarization of microglia toward a less inflammatory phenotype. As an epigenetic anti-inflammatory agent, curcumin has been hypothesized to mediate its effects, in part, through the suppression of TREM-1. Yuan et al. demonstrated that curcumin downregulates TREM-1 expression both in vitro, using primary bone marrow-derived macrophages (BMDMs), and in vivo, in the lungs of septic mice. This suppression was attributed to curcumin’s ability to inhibit p65 binding to the TREM-1 promoter and reduce p300 activity, leading to hypoacetylation of histones 3 and 4 at lysine residues within the TREM-1 promoter region. In a more recent study, Nguyen et al. explored in vitro and in silico neuroprotective mechanisms of curcumin in the context of 1,2-diacetylbenzene (DAB)-induced neuroinflammation, with a focus on TREM-1 suppression. Curcumin was found to inhibit both the TREM-1/DAP12/NLRP3/caspase-1/IL-1β and TLR4/NFκB signaling pathways induced by DAB. Additionally, curcumin reduced reactive oxygen species (ROS), advanced glycation end products (AGEs), hyperphosphorylation, GSK-3β activity, and β-amyloid accumulation in microglial BV2 cells, while simultaneously enhancing the expression of Nrf2. In silico analysis identified key genes and transcription factors, TNF, IL6, NFκB1, IL10, IL1B, MTF1, and ZNF267, involved in DAB-induced cognitive impairment and curcumin’s inhibitory effects. Additionally, three miRNAs (*hsa-miR-26a-5p*, *hsa-miR-203a-3p*, and *hsa-miR-155-5p*) were linked to DAB pathology and targeted by curcumin. Together, these results suggest curcumin may counteract neuroinflammation and cognitive deficits, supporting its potential as a treatment for inflammatory conditions like sepsis-related cognitive impairment [132,133,134,135].

**Doxycycline**: Doxycycline (SDD), a member of the tetracycline class of antibiotics, is widely recognized for its anti-inflammatory properties. SDD, at submicromole doses typically ranging from 2 to 10 µg mL^−1^, are frequently utilized as an adjunct to conventional therapy in the management of chronic periodontitis. Periodontitis is often associated with pathogenic bacteria such as *Porphyromonas gingivalis* and *Aggregatibacter actinomycetemcomitans*. *P. gingivalis* is predominantly linked to chronic periodontitis in adults, whereas *A. actinomycetemcomitans* is more commonly implicated in aggressive forms of the disease, particularly in younger individuals. SDD, at subantimicrobial concentrations, does not disrupt the gut microbiota but effectively suppresses the production of pro-inflammatory cytokines in monocytes exposed to periodontal pathogens such as *A. actinomycetemcomitans*. Additionally, studies have demonstrated that *P. gingivalis* modulates the TREM-1/DAP12 signaling pathway in monocytic cells. Specifically, *P. gingivalis* at a multiplicity of infection (MOI) of 100 upregulates TREM-1 gene expression and *s*TREM-1 secretion without significantly altering DAP12 expression. Bostanci et al. [75,77,136] investigated the impact of SDD on *P. gingivalis*-induced TREM-1 expression and secretion in the myelomonocytic cell line MonoMac-6. Their findings revealed that *P. gingivalis* significantly increased both TREM-1 gene expression and *s*TREM-1 release after 24 h of exposure. However, pretreatment with SDD for 4 h effectively abolished TREM-1 expression and secretion. These results suggest that the clinical efficacy of SDD as an adjunctive treatment for periodontal disease is partially mediated through its ability to inhibit pathogen-induced TREM-1 activation, thereby attenuating the inflammatory response associated with periodontitis [75,77,136].

**VJDT**: VJDT has recently been identified as a TREM-1 inhibitor, although high concentrations (50 µM) were required. This compound was discovered using virtual screening combined with receptor-based molecular docking on a variety of NCI small molecule libraries. Chemically, VJDT is a substituted *2,3,3a,4,7,7a*-hexahydro-*4,7*-(epiminocarbonyl)isoindole analog, employed to assess the effects of TREM-1 inhibition on tumor progression. To investigate the impact of VJDT, Ajith et al. [104] utilized melanoma patient-derived xenograft (PDX) tumors as well as mouse models of tumor growth, induced by subcutaneous injections of B16F10 murine melanoma cells or MCA205 fibrosarcoma cells. VJDT treatment effectively slowed tumor growth in both models by promoting the infiltration of CD8^+^ PD-1^+^ T cells into the tumor microenvironment. Additionally, VJDT therapy in patient-derived melanoma xenograft tumors downregulated key oncogenic signaling pathways involved in cell proliferation, migration, and survival. TREM-1 inhibition with VJDT not only reduced tumor progression but also enhanced the effectiveness of PD-1 immune checkpoint blockade. Therefore, VJDT, as a TREM-1 inhibitor, shows promise as a novel cancer therapeutic agent [104].

## 6. Applications of Targeting TREM-1 Signaling in Human Studies and Clinical Trials

TREM-1 has emerged as a critical regulator of various pathophysiological processes, including angiogenesis, pneumonia, cardiovascular diseases, carcinogenesis, wound healing, etc. The biological functions of TREM-1 are modulated by its binding affinity to specific ligands, making it an attractive target for drug development. Therapeutic modulation of TREM-1 has shown promise in managing a range of diseases, for example, diabetes, cancer, chronic kidney disease, cardiovascular disorders, and neurological conditions [5,19,54,108,122,123].

Extensive basic, preclinical, and clinical research have advanced the development of TREM-1 targeted therapies in the treatment of disease. While some non-selective TREM-1 inhibitors have demonstrated potential in treating some conditions, their clinical utility remains constrained by off-target effects and suboptimal pharmacokinetic profiles. Therefore, to advance precision medicine approaches, it is critical to identify TREM-1 pathway alterations and associated molecular modifications that can predict therapeutic response. Furthermore, a deeper understanding of the role of TREM-1 involvement in the emergence of drug resistance may inform the rational design of combination regimens. Integrating selective TREM-1 inhibitors with other targeted therapies could potentiate clinical efficacy and overcome resistance mechanisms, representing a strategic direction for future therapeutic development.

A major cause of mortality, apart from early organ failure, is secondary infection-induced multiple organ dysfunction. The accurate diagnosis and treatment of such conditions remain formidable challenges for clinicians. Since most of these conditions are associated with inflammation, several clinical investigations have explored TREM-1 as a potential therapeutic target, yielding promising findings. COVID-19 infection has been characterized by a hyperinflammatory state, endotheliopathy, and coagulopathy, with TREM-1 playing a key role in exacerbating these processes [137]. A clinical trial (NCT04544891) was conducted to evaluate the relationship between TREM-1 pathway activation, disease progression, and clinical outcomes in hospitalized COVID-19 patients. Recent studies have identified *s*TREM-1 as a key biomarker in COVID-19, with elevated levels correlating strongly with disease severity and poor outcomes. High *s*TREM-1 levels were predictive of complications, ICU admission, and mortality. The TREM-1 pathway inhibitor, nangibotide, was shown in a clinical trial to enhance clinical condition and reduce mortality among patients with severe COVID-19 who had high *s*TREM-1 levels. Nevertheless, because of slow patient recruitment, the trial was terminated early, and no significant difference was observed in the secondary endpoint of 28-day all-cause mortality between nangibotide and placebo. These results emphasize the critical involvement of TREM-1 in COVID-19–associated hyperinflammation and underscore its promise as both a prognostic indicator and a therapeutic target [138,139,140].

TREM-1 has been studied in relation to clinical outcomes in sepsis and other inflammatory conditions. A clinical study (NCT0140424) evaluated associations between *s*TREM-1 levels and sepsis outcomes in 80 patients and 80 control subjects. Non-survivors showed higher circulating *s*TREM-1 concentrations compared with survivors, and regression analysis revealed associations between *s*TREM-1, rs2234237, and APACHE II polymorphisms and disease outcome. Another trial (NCT00976157) examined *s*TREM-1 concentrations in bronchoalveolar lavage (BAL) fluid from patients with ventilator-associated pneumonia (VAP), assessing their relationship with disease progression and clinical course. Similarly, a study in patients with severe acute pancreatitis (NCT01193413) measured *s*TREM-1 levels in fine-needle aspiration (FNA) fluid to investigate correlations with secondary infections in necrotic tissue. Collectively, these studies report associations between elevated *s*TREM-1 levels and adverse inflammatory states, though their prognostic value remains to be validated [46,141]. A summary of these studies, along with other clinical trials of TREM-1 inhibitors, is depicted in Table 5. The multifaceted role of TREM-1 across various disease states underscores its potential as a biomarker and therapeutic target. These findings highlight the need for continued research into TREM-1-targeted drug development, which could lead to innovative treatment approaches for inflammatory, infectious, and immune-mediated disorders.

## 7. Conclusions and Future Research

Inhibiting TREM-1 presents a promising strategy for treating inflammatory diseases. Primarily found on myeloid cells, including neutrophils and monocytes, TREM-1 acts as a potent enhancer of inflammation by boosting the production of pro-inflammatory cytokines in response to microbial or non-infectious triggers. Overactive TREM-1 signaling has been associated with conditions such as sepsis, acute lung injury, inflammatory bowel disease, rheumatoid arthritis, and inflammation related to cancer. In animal studies, blocking TREM-1 using agents like LR12, GF9 peptides, decoy receptors, or monoclonal antibodies has been shown to reduce inflammation, limit tissue damage, and improve survival. These findings emphasize TREM-1 inhibition as a potential therapeutic approach to rebalance immune responses without causing broad immunosuppression.

TREM-1 serves as a critical regulator in a range of diseases, including neurological, cardiovascular, cancer-related, and inflammatory conditions. Although its importance is well recognized, further research is essential to fully understand the complexities of TREM-1 signaling and its specific molecular functions in inflammatory conditions due to other insults, such as cerebral ischemia and hearing loss linked to noise exposure or aminoglycoside antibiotics.

At present, most therapeutic approaches focus on inhibiting TREM-1 activation. While early-stage research has shown encouraging outcomes, TREM-1-targeted treatments have yet to reach routine clinical use. Nangibotide, a peptide-based formulation of LR-12, is undergoing clinical trials for sepsis treatment. However, its peptide nature confers it an extremely short half-life of about two minutes, posing challenges for drug development due to poor pharmacokinetics and the potential for off-target/unwanted effects, especially with long-term use. This highlights the need for (1) the development of new strategies to enhance pharmacokinetic stability of peptide-based drugs and (2) the development of small-molecule inhibitors that selectively target TREM-1, offering better stability and safety profiles without the drawbacks associated with peptide-based drugs.

Recent research has focused on advanced drug delivery systems, such as packaging TREM-1 inhibitor GF9 within artificial high-density lipoprotein nanoparticles, to improve precision and safety. These targeted methods may enhance the clinical effectiveness of TREM-1 inhibitors. Nonetheless, more studies are required to understand how specific alterations in TREM-1 signaling influence disease progression and treatment outcomes.

Several other strategies may sustain LR12 or GF9 bioavailability, including PEGylation to reduce renal clearance, lipidation or albumin-binding motifs to promote reversible plasma protein association, and Fc-fusion constructs to leverage the neonatal Fc receptor recycling pathway [142,143,144,145]. Incorporation of D-amino acids or cyclization can improve proteolytic resistance [146,147], while depot or nanoparticle delivery systems may sustain local release and reduce dosing frequency [113]. In parallel, small-molecule TREM-1 modulators offer complementary advantages such as oral bioavailability, broader tissue penetration, and scalable manufacturing [104]. As peptide-based therapeutics advance, emphasis should be placed on monitoring safety and immunogenicity profiles rather than assuming inherent peptide toxicity, ensuring translational readiness for systemic inflammatory indications.

Combining TREM-1 inhibitors with other immune-modulating agents, such as cytokine blockers or checkpoint inhibitors, may offer new treatment possibilities for cancer and complex inflammatory disorders. Toll-like receptor (TLR) antagonists might indirectly affect TREM-1 signaling since TLR ligands are known to increase TREM-1 expression. However, their use in modulating TREM-1 remains largely unexplored. Although levels of TREM-1 and *s*TREM-1 have been investigated for diagnostic purposes, other TREM family proteins have not been thoroughly evaluated as biomarkers. Due to the diverse roles of TREM receptors, developing selective inhibitors and activators is challenging and raises concerns about potential off-target effects.

Since TREM-1 activation is context-specific and tightly regulated, a deeper understanding of how various ligands trigger TREM-1 in different disease settings is essential. This knowledge will help in designing more precise and effective therapies. Moving forward, a multidisciplinary approach combining systems biology, pharmacology, and immunology will be key to unlocking the full therapeutic potential of TREM-1 inhibition in inflammatory diseases.

## Figures and Tables

**Table 1:** Role of ligands/activators on TREM1 activation and their biological effects.

Ligand	Evidence for Interaction with TREM-1	Biological Response via TREM-1	Remarks	Ref.
LPS	LPS is not a direct ligand but acts upstream to induce TREM-1 expression; TREM-1 amplifies LPS responses.	Its stimulation provides TREM-1 upregulation, clustering (multimerization), enhanced Ca^2+^ flux, ROS, cytokine production.	The contribution of TREM-1 is considered an amplifier rather than the primary receptor.	[5,45,46]
PGLYRP1 (Tag7)	Available in 3 forms: 17, N15 and N9. Identified as a ligand capable of binding *s*TREM-1 and activating TREM-1 in reporter assays/cell culture.	Induces phosphorylation of SYK, release of IL-6, *s*TREM-1, and amplifies inflammation.	Binding affinity for TREM-1; 3 peptides 17 Kd = 1.3 nM, N15 Kd = 7.4 nM and N9 Kd = 9.7 nM. The activation requires peptidoglycan crosslinking.	[31]
HMGB1	Shown via cross-linking/immunoprecipitation to bind TREM-1	Necrotic lysates with HMGB1 stimulates TREM-1 dependent IL-1β, IL-6, TNF, IFN, and via TREM-1 signaling	Binding affinity for TREM-1 Kd = 35.4 µM. Often acts in concert with other co-activators (nucleic acids, DNA, other DAMPs) to fully engage inflammatory receptors.	[47,48]
Actin	Recombinant actin (or actin from necrotic/platelet) binds the TREM-1 extracellular domain and enhances inflammatory responses; effect absent in TREM-1 knockout mice.	Co-injection of actin in mice enhances inflammatory responses in wild-type but not in TREM-1^–/–^ mice; synergizes with LPS.	The physiological relevance of extracellular actin as a TREM-1 ligand under sterile injury or infection is still under investigation.	[37]
HSP70	HSP70 has been shown to bind TREM-1 (or *s*TREM-1) and induce cytokine mRNAs in monocytes (e.g., TNFα, IFNγ).	Activation increases TNF-α, IFN-γ mRNA in monocytes, IL-2 secretion by PBMCs; stimulates maturation/ activation of cytotoxic lymphocytes in co-culture assays.	Binding affinity of HSP70 N7 peptide for TREM-1 Kd = 1.6 nM. The strength and conditions (e.g., necrotic lysate, co-factors) affect activation.	[39]
eCIRP	Demonstrated as a bona fide ligand: via surface plasmon resonance, FRET, macrophage binding. Blocking TREM-1 (or TREM-1 knockouts) reduces eCIRP-induced inflammation.	eCIRP binding to TREM-1 triggers DAP12 → Syk → downstream signaling; in vivo, blocking using peptide M3 or LP17 reduces systemic inflammation and tissue injury in sepsis/ALI models.	Binding affinity for TREM-1 Kd = 117 nM. Because eCIRP is a more recently characterized ligand, more studies are needed to establish its relative importance among TREM-1 ligands.	[40,41,49]
CD177	CD177 is described as an endogenous TREM-1 ligand	TREM-1 activation increases NETs and IL-22 in CD177^+^ neutrophils, contributing to bacterial clearance and epithelial barrier protection.	Direct molecular binding interface (CD177-TREM-1) is not well characterized; much of the evidence is indirect or inferred.	[43]

**Table 2:** Summary of TREM-1, TLR4, and NLRP3 activation priority.

Disease	Primary Trigger	Activation Priority	TREM-1 Role
Sepsis	LPS	TLR4 → TREM-1 → NLRP3	Amplifier of TLR4
Neuro inflammation	DAMPs	TLR4 → TREM-1 and NLRP3 (parallel)	Synergistic inflammation
RA	Endogenous ligands	TLR4 → TREM-1 → NLRP3	Joint inflammation amplifier
Hepatic/ Renal IRI	DAMPs	TLR4 + TREM-1 → NLRP3 (cooperative)	Sterile injury amplifier
Liver fibrosis	Chronic inflammation	TLR4/NLRP3 → TREM-1	Late-stage tumor promoter
COPD	Cigarette smoke	TREM-1 ↑ → TLR4 → NLRP3	Early driver & amplifier
IBD	Microbial/ DAMPs	TLR4/NOD2 → TREM-1 → NLRP3	Amplifier, severity marker

→ indicates signaling cascade flow; ↑ indicated upregulation.

**Table 5:** Clinical trials exploring modulation of TREM-1 signaling in disease.

NCT (Number)	Status	Condition/Study Type	Intervention or Measure	Location(s)
NCT02873949	Completed	Periodontitis (Observational biomarker study)	Crevicular and saliva samples; etiologic treatment	Central Hospital, Nancy, France
NCT01490424	Completed	Sepsis (Genetic association study)	Analysis of *Trem-1* gene polymorphisms	Chinese PLA General Hospital, Beijing, China
NCT04948840	Not Yet Recruiting	Radiation-induced mammary fibrosis (Observational biomarker study)	Assessment of TREM-1 expression and activation	National Taiwan University Hospital, Taipei, Taiwan
NCT04544891	Not Yet Recruiting	COVID-19 (Observational biomarker study)	Blood sampling for TREM-1 pathway activation	CHRU Limoges & Central Hospital Nancy, France
NCT05020496	Not Yet Recruiting	UV-induced immune suppression (Mechanistic study)	Diphenyl cyclopropenone (DPCP) challenge	University of Alabama at Birmingham, USA
NCT00645619	Withdrawn	Viral and bacterial pneumonia (Diagnostic biomarker study)	TREM-1 protein assay	Children’s Medical Center, Dallas, Texas, USA
NCT01903668	Withdrawn	Sepsis (Ligand identification study)	Endogenous TREM-1 ligand analysis	Centre Hospitalier Universitaire Dijon, France
NCT00976157	Completed	Ventilator-associated pneumonia (Observational biomarker study)	Serial *s*TREM-1 measurement in BAL fluid	Mackay Memorial Hospital, Taipei, Taiwan
NCT01193413	Completed	Severe acute pancreatitis (Observational biomarker study)	Measurement of *s*TREM-1 in FNA fluid	Changhai Hospital, China
NCT01410578	Completed	Sepsis/Bacteremia (Diagnostic biomarker study)	Measurement of *s*TREM-1, PCT, CRP	Chinese PLA General Hospital, Beijing, China
NCT03158948	Completed	Septic shock (Interventional, TREM-1 blockade)	Drug: Motrem (anti-TREM-1 mAb) compared with placebo	Multicenter (Belgium, France, The Netherlands, Spain)

## Data Availability

No new data were created or analyzed in this study. Data sharing is not applicable to this article.

## Abbreviations

The following abbreviations are used in this manuscript:

TREM-1	Triggering Receptors Expressed on Myeloid Cells-1
DAP12	DNAX activation protein 12
ITAM	Immunoreceptor tyrosine-based activation motif
SH2	Src homology 2
ZAP70	Zeta-chain-associated protein kinase 70
SYK	Spleen Tyrosine Kinase
PI3K	Phosphoinositide 3-kinase
ERK	Extracellular signal-regulated kinase
PRRs	Pathogen recognition receptors
DAMPs	Damage-associated molecular patterns
PAMPs	Pathogen-associated molecular patterns
IRAK-4	IL-4 receptor-associated kinase-4
NFκB	Kappa-light-chain-enhancer of activated B cells
LPS	lipopolysaccharide
CRE	cAMP response element
CREB	CRE-binding protein
PKA	Protein kinase A
AP-1	Activator protein-1
Nrf2	Nuclear factor erythroid 2-related factor 2
PGE2	Prostaglandin E2
NLR	Nucleotide-binding oligomerization domain(NOD)-like receptors
PMNs	Polymorphonuclear neutrophils
PGLYRP1	Peptidoglycan Recognition Protein 1
HMGB1	High Mobility Group Box 1
Hsp70	Heat Shock Protein 70
IFN-γ	Interferon-gamma
TNF-α	Tumor necrosis factor-alpha
CIRP	Cold-Inducible RNA-Binding Protein
IL	Interleukin
BALF	Bronchoalveolar lavage fluid
CVDs	Cardiovascular diseases
CAD	Coronary artery disease
AMI	Atherosclerotic myocardial infarction
SAH	Subarachnoid hemorrhage
CSF	Cerebrospinal fluid
BBB	Blood–brain barrier
Aβ	Amyloid-beta
RA	Rheumatoid arthritis
M-CSF or CSF-1	Macrophage colony-stimulating factor
CIA	Collagen-induced arthritis
ROS	Reactive oxygen species
TOMM40	Translocase of the outer mitochondrial membrane 40
RANK	Receptor activator of NFκB
OPG	Osteoprotegerin
APDC	Atherosclerosis, periodontitis, diabetes, and cancer
HCC	Hepatocellular carcinoma
HSCs	Hepatic stellate cells
TGF-β1	transforming growth factor-beta 1
SMA	smooth muscle actin
NAFLD	Nonalcoholic fatty liver disease
TLT-1	Triggering receptor expressed on myeloid cells-like transcript 1
AAA	Abdominal aortic aneurysm
SCHOOL peptide	Signaling Chain HOmoOLigomerizationpeptide
BTK	Bruton’s tyrosine kinase
BMDMs	Bone marrow-derived macrophages
DAB	Diacetylbenzene
PDX	Patient-derived xenograft
VAP	Ventilator-associated pneumonia
